# Genome-wide expression profiles of *Pyropia haitanensis* in response to osmotic stress by using deep sequencing technology

**DOI:** 10.1186/s12864-015-2226-5

**Published:** 2015-11-26

**Authors:** Li Wang, Yunxiang Mao, Fanna Kong, Min Cao, Peipei Sun

**Affiliations:** Key Laboratory of Marine Genetics and Breeding (Ocean University of China), Ministry of Education, College of Marine Life Science, Ocean University of China, Qingdao, 266003 China; Institute of Plant Resources, Dalian Nationalities University, Dalian, 116600 China; Laboratory for Marine Biology and Biotechnology, Qingdao National Laboratory for Marine Science and Technology, Qingdao, 266200 China

**Keywords:** *Pyropia haitanensis*, Osmotic stress, Dehydration, Rehydration, DGE

## Abstract

**Background:**

*Pyropia haitanensis* is an economically important marine crop grown in harsh intertidal habitats of southern China; it is also an excellent model system for studying mechanisms of stress tolerance. To understand the molecular mechanisms underlying osmotic tolerance and adaptation to intertidal environments, a comprehensive analysis of genome-wide gene expression profiles in response to dehydration and rehydration in *Py. haitanensis* was undertaken using digital gene expression profile (DGE) approaches combined with *de novo* transcriptome sequencing.

**Results:**

RNA-sequencing of the pooled RNA samples from different developmental phases and stress treatments was performed, which generated a total of 47.7 million clean reads. These reads were *de novo* assembled into 28,536 unigenes (≥200 bp), of which 18,217 unigenes (63.83 %) were annotated in at least one reference database. DGE analysis was performed on four treatments (two biological replicates per treatment), which included moderate dehydration, severe dehydration, rehydration, and normal conditions. The number of raw reads per sample ranged from 12.47 to 15.79 million, with an average of 14.69 million reads per sample. After quality filtering, the number of clean reads per sample ranged from 11.83 to 15.04 million. All distinct sequencing reads were annotated using the transcriptome of *Py. haitanensis* as reference. A total of 1,681 unigenes showed significant differential expression between moderate dehydration and normal conditions, in which 977 genes were upregulated, and 704 genes were downregulated. Between severe dehydration and normal conditions, 1,993 unigenes showed significantly altered expression, which included both upregulated (1,219) and downregulated genes (774). In addition, 1,086 differentially expressed genes were detected between rehydration and normal conditions, of which 720 genes were upregulated and 366 unigenes were downregulated. Most gene expression patterns in response to dehydration differed from that of rehydration, except for the synthesis of unsaturated fatty acids, several transcription factor families, and molecular chaperones that have been collectively implicated in the processes of dehydration and rehydration in *Py. haitanensis*.

**Conclusions:**

Taken together, these data provide a global high-resolution analysis of gene expression changes during osmotic stress that could potentially serve as a key resource for understanding the biology of osmotic acclimation in intertidal red seaweed.

**Electronic supplementary material:**

The online version of this article (doi:10.1186/s12864-015-2226-5) contains supplementary material, which is available to authorized users.

## Background

The marine red alga *Pyropia* is one of the most economically important mariculture crops. It has an annual production of at least 120,000 tons (dry weight), which is estimated to be worth at least US$1.3 billion per year [1, 2]. For hundreds of years, this crop has been cultivated in East Asian countries such as China, Korea, and Japan, of which it is currently recognized as one of its largest aquaculture industries [3]. *Pyropia haitanensis* Chang et Zheng, an endemic species naturally distributed and widely cultivated along the coasts of South China, has comprised 75 %–80 % of the total production of cultivated *Pyropia* spp. in China [4].

As sessile organisms that inhabit in the intertidal zones of rocky coasts, the thallus of *Py. haitanensis* is totally submerged in water during high tide, but is exposed to the air during low tide. Therefore, this crop is constantly exposed to fluctuating and extreme abiotic conditions such as cyclic changes in light levels, abrupt temperature changes, and repeated desiccation/rehydration due to the turning tides. Consequently, *Py. haitanensis* experiences far more rapid and severe water loss during low tide, and unavoidably suffers from dramatic changes of osmotic potential, which were largely different from the environmental stresses experienced by desiccation-tolerant land plants. *Py. haitanensis* can tolerate extreme water loss (up to 70 %) and dramatic changes in osmotic potential, consistent with other *Pyropia*/*Porphyra* species [5–8]. Therefore, *Py. haitanensis* is considered an ideal model systerm for investigating the mechanisms of osmotic acclimation in intertidal seaweed.

Several studies have elucidated that anatomical properties, as well as physiological and biochemical changes allow *Pyropia* and *Porphyra* species to tolerate osmotic stress, which in turn allow them to thrive in the intertidal zone. For example, blade cells secrete a cell wall that is an agar-like sulfated galactan disaccharide (porphyran) with xylan microfibrils and proteoglycan elements [9]; this hydrophilic wall could reduce the rate of water and mineral loss from *Pyropia* and *Porphyra* species during emersion. Metabolic analyses showed that an increase in O-α-D-galactopyr-anosyl-(1→2)-glycerol (= floridoside) occurs under high salinity stress, suggesting that it acts as a compatible solute in reducing cytoplasmic and membrane damage [10]. Abe et al. [11] reported that *Pyropia dentata*, a species that thrives at the highest intertidal level, can fully recover its photosynthetic activity after desiccation at a water potential of -158 MPa. Several protective proteins are secreted and accumulate during osmotic stress, which have been shown to play a role in cellular protection during stress. For example, Contreras-Porcia et al. [12] observed that in *Porphyra columbina*, the activity of a diverse range of antioxidant enzymes increased during desiccation, whereas this activity diminished to near basal levels during rehydration. These intracellular proteins provide protection against osmotic stress by stabilizing membranes and organelles, as well as counteracting oxidative stress. Furthermore, high levels of a dehydrin-like protein with a molecular weight of 17 kDa, which is extremely hydrophilic under dehydrative conditions and thermally stable, was detected in *Porphyra umbilicalis*, but not *Pyropia yezoensis*, which suggests that this protein plays a key role in the observed superior desiccation tolerance in the former species [13]. In addition, expression of cyclophilin (*Ph*CYP18) was shown to be dysregulated in the blades of *Py. haitanensis* under high salt stress, strong irradiance stress, and multifactorial stress compared to blades under normal conditions, thus suggesting that cyclophilin actively responded to stress situations and induced high stress tolerance [14].

In the advent of next-generation sequencing (NGS), whole genome-wide expression profiling have become a rapid and efficient method for identifying genes and specific metabolic processes involved in stress response, especially in organisms whose genomes have not been fully sequenced. Small-scale cDNA sequencing projects, cDNA microarrays, and *de novo* transcriptome sequencing have been performed in several stress-tolerant intertidal algae, including some *Pyropia* species [15–23]. Moreover, plastid and mitochondrial genomes of several *Pyropia* species have recently been sequenced [24–26]. In addition, the draft genome sequence of *Py. yezoensis* has been determined using NGS [27], whereas whole genome sequencing projects of other *Pyropia* species (i.e. *Py. haitanensis*) have not been completed. Although the *Pyropia* species may be a source of genetic determinants for osmotic acclimation, defense response-related gene discovery efforts in marine algae have been limited, and the molecular mechanisms involved in osmotic acclimation remain unknown to date.

To understand the molecular mechanisms underlying osmotic acclimation and adaptation to intertidal environments, a comprehensive analysis of genome-wide gene expression profiles in response to dehydration and rehydration in *Py. haitanensis* was performed. Because the genome sequence of *Py. haitanensis* is not yet available, deep expression profiling is feasible by large-scale sequencing of transcripts from eight hydrated and dehydrated *Py. haitanensis* algae samples using Illumina HiSeq 2000 platform. This sequencing dataset and analysis results will serve as a valuable resource for identifying the key genes and pathways involved in the response to osmotic stress in *Py. haitanensis*. The findings of the present study will lay the foundation for elucidating the molecular mechanisms of osmotic acclimation and provide useful information for the genetic breeding of *Py. haitanensis*.

## Results

### Global transcriptome assembly

Due to the limited genome information on *Py. haitanensis*, to obtain a global transcriptome of *Py. haitanensis*, a cDNA library was constructed from an equal mixture of RNA isolated from different developmental phases and treatments (Additional file 1: Table S1). The library was sequenced using the Illumina HiSeq 2000 platform. A total of 53.46 million raw reads from the sequencing library were obtained. After quality filtering, a total of 47.76 million (about 89.35 % of the raw reads) clean reads were obtained, corresponding to 4.78 G bases. The GC content of the transcriptome was 64.93 %. An overview of the sequencing is presented in Additional file 1: Table S1.

Using the Trinity *de novo* assembly program [28], all clean reads were assembled into 31,838 transcripts, with an N50 length of 956 bp and an average length of 657 bp (Additional file 1: Table S1). The transcripts were subjected to cluster analysis. From these transcripts, a total of 28,536 unigenes were obtained, with an N50 length of 827 bp and an average length of 607 bp (Additional file 1: Table S1). The length statistics of assembled transcripts and unigenes revealed that 9,398 unigenes (32.9 %) were > 500 bp, and 3,854 unigenes (13.5 %) were > 1 kb (Additional file 1: Table S1). These results demonstrated the effectiveness of Illumina sequencing in rapidly capturing a large portion of the transcriptome. As expected, for a randomly fragmented transcriptome, there was a positive relationship between the length of a given unigene and the number of reads assembled into it (Additional file 2: Figure S1).

### Function annotation of transcriptome unigenes

To assign accurate annotation information to all unigenes, multiple databases were interrogated, including NCBI non-redundant protein (Nr) database, NCBI non-redundant nucleotide sequence (Nt) database, the manually annotated and curated protein sequence (Swiss-Prot) database, Protein family (Pfam), euKaryotic Ortholog Groups (KOG), Gene Ontology (GO), and Kyoto Encyclopedia of Genes and Genomes (KEGG) database. The overall functional annotation is presented in Table 1. A total of 18,217 unigenes (63.83 %) were annotated in at least one database. Among these, 9,114 (31.93 %) had significant matches in the Nr database, whereas 7,562 (26.49 %) unigenes showed similarity to proteins in the Swiss-Prot database. In addition, 13,972 (48.96 %) and 4,952 (17.35 %) unigenes were successfully annotated in the GO and KEGG databases, respectively.

**Table 1:** Statistics of functional annotation of the *Py. haitanensis* transcriptome

Annotated databases	Number of annotated unigenes	Percentage of annotated unigenes
Nr	9,114	31.93 %
Nt	1,705	5.97 %
Swiss-Prot	7,562	26.49 %
Pfam	13,238	46.39 %
KOG	12,228	42.85 %
GO	13,972	48.96 %
KEGG	4,952	17.35 %
Annotated in all databases	1,148	4.02 %
Annotated in at least one database	18,217	63.83 %
Total queries/unigenes	28,536	100.0 %

### Sequencing and annotation for DGE libraries

To characterize the digital gene expression profiles (DGE) involved in *Py. haitanensis* response to dehydration/rehydration, a total of eight RNA samples generated from two biological replicates under the four treatments [normal conditions (CON), moderate water loss (MWL), severe water loss (SWL), and rehydration (REH)] were subjected to DGE sequencing analysis by using a Illumina HiSeq 2000 sequencing platform, respectively (Table 2). In total, 117.48 million raw reads were generated from the eight samples. The number of raw reads per sample ranged from 12.47 to 15.79 million, with an average of 14.69 million reads per sample. About 95 % of the raw reads passed the quality filters, resulting in a total of 111.22 million clean reads. The number of clean reads in each sample ranged from 11.83 to 15.04 million.

**Table 2:** Summary of sequencing results for eight DGE libraries

Sample	Raw reads	Clean reads	Clean bases	Error (%)	Q20 (%)	Q30 (%)	GC (%)
MWL_1	13,868,151	13,050,154	1.31G	0.07	93.65	83.42	65.63
MWL_2	14,979,652	14,240,476	1.42G	0.07	94.06	84.08	65.10
SWL_1	14,163,997	13,279,452	1.33G	0.08	93.40	82.94	65.61
SWL_2	15,596,177	14,781,653	1.48G	0.07	94.12	84.16	65.96
REH_1	14,980,200	14,168,609	1.42G	0.07	93.94	83.60	65.87
REH_2	12,471,254	11,830,575	1.18G	0.07	94.13	84.14	65.87
CON_1	15,792,640	15,041,472	1.5G	0.07	94.19	84.03	66.32
CON_2	15,631,909	14,827,844	1.48G	0.07	93.82	83.18	66.05

Clean reads from the eight samples were mapped to the global transcriptome of *Py. haitanensis* and obtained from the annotation information of each sample using RSEM software [29]. The number of mapped clean reads in each sample ranged from 11.50 to 14.56 million, accounting for 96.78 %–97.74 % of the total clean reads (Additional file 3: Table S2). In addition, the number of read-mapped unigenes ranged from 19,843 to 21,954 (Additional file 3: Table S2). To evaluate the reproducibility of DGE library sequencing, Pearson correlation analysis was performed on every two replicates. A series of scatterplots comparing the normalized read counts (log transformed) for the replicate libraries in each treatment are shown in Additional file 4: Figure S2. The results showed that the square of the Pearson correlation coefficient (R^2^) between two replicate libraries in each treatment was > 0.91, indicating the reliability as well as operational stability of the experimental results.

### Analysis of differentially expressed genes

Read count data was used to identify the differentially expressed genes (DEGs) among various treatments by using DESeq packages [30]. A total of 1,681, 1,993, and 1086 differentially expressed genes were identified (adjusted *P*-value of < 0.05) between MWL and CON, SWL and CON, and REH and CON, respectively.

**Fig. 1:**
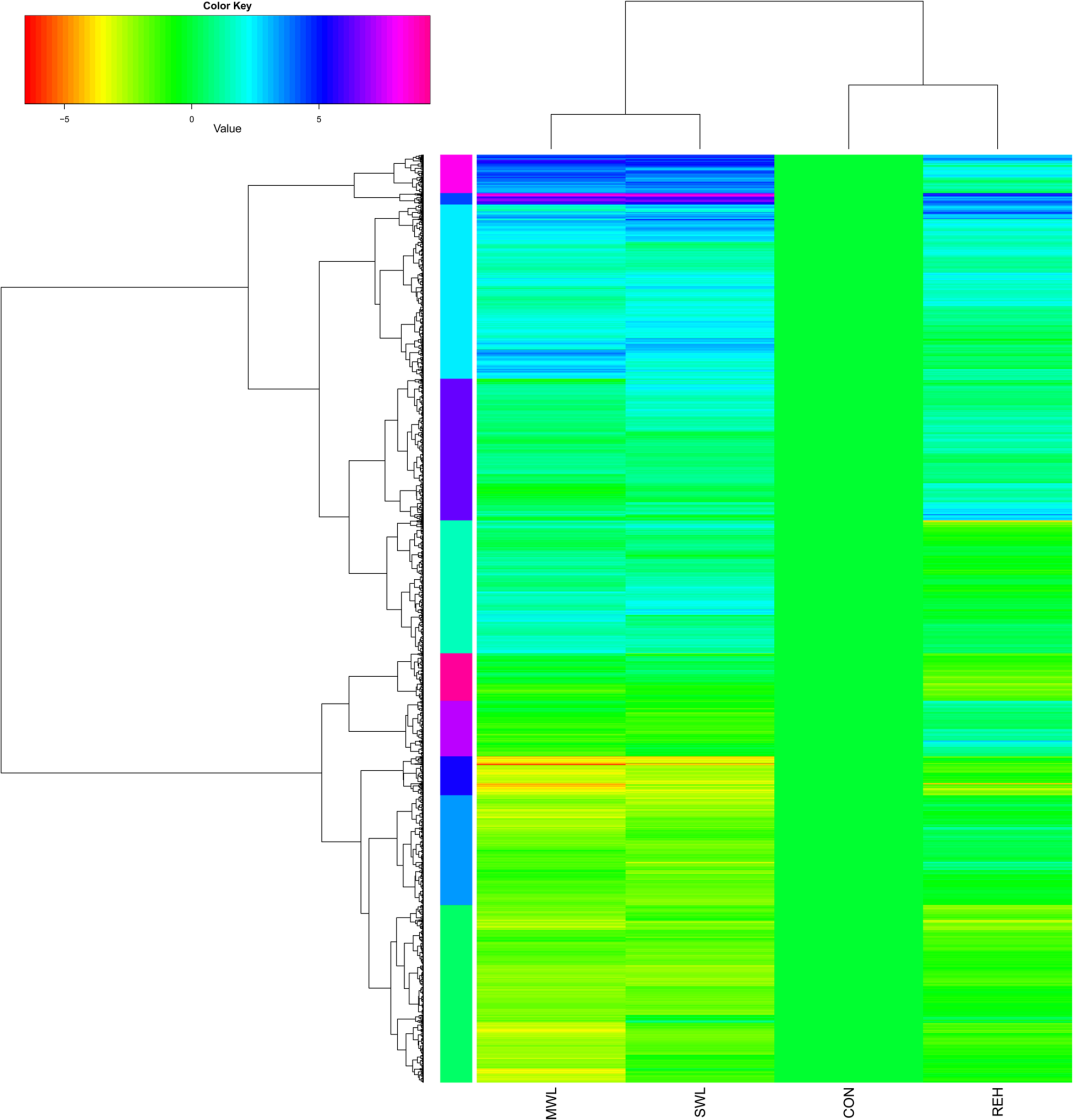
Expression profile clustering of 3,569 DEGs of pairwise comparison among four different treatments. Colors indicate expression values scaled to standard deviations and centered at the normal intensity level (Z-score). Purple indicates increased expression and red decreased, relative to the normal conditions. The 10 different colored bars indicate unigenes in 10 subclusters in our data

**Fig. 2:**
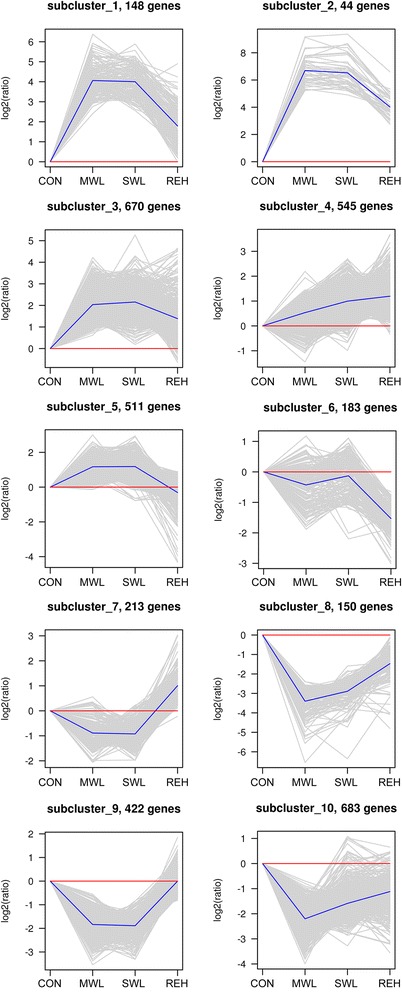
Expression graphs of subclusters identified by the hierarchical cluster analysis. Ten subclusters represent expression signatures of 3,569 differentially expressed genes of *Py. haitanensis* using four different treatments. The *x*-axis represents the treatments. The *y*-axis represents the value of the relative expression level [log_2_ (ratio)]. The gray line represents the value of the relative expression level, the blue line represents the mean value of the relative expression level, and the red line is the reference

A hierarchical cluster analysis of normalized expression values for the 3,569 DEGs from pairwise comparisons among the four treatments (MWL *vs*. CON, SWL *vs*. CON, MWL *vs*. SWL, REH *vs*. CON, MWL *vs*. REH, and SWL *vs*. REH) was performed (Fig. 1). The analysis showed that the gene expression pattern in the MWL treatment was similar to that of the SWL treatment, whereas it was markedly distinct from the REH treatment. Furthermore, the 3,569 unigenes were divided into 10 subclusters based on their expression modulation, representing 10 different expression patterns (Fig. 2). In subcluster 1, the unigenes were significantly enriched in the pathway of aminoacyl-tRNA biosynthesis, DNA replication, mismatch repair, porphyrin and chlorophyll metabolism, and nucleotide excision repair. In subclusters 2 and 3, no significant enrichment was detected. Subcluster 4 was significantly enriched with the pathway of pyrimidine metabolism, folate biosynthesis, and RNA polymerase. In subcluster 5, the unigenes were significantly enriched in the pathway of carbon fixation in photosynthetic organisms, microbial metabolism in diverse environments, pentose phosphate pathway, fructose and mannose metabolism, glycolysis/gluconeogenesis, and biosynthesis of ansamycins. In subcluster 6, the unigenes were significantly enriched in the pathway of DNA replication, mismatch repair, base excision repair, nucleotide excision repair, cell cycle, and non-homologous end-joining. In subcluster 7, protein processing in endoplasmic reticulum was significantly enriched. In subclusters 8, 9, and 10, no significant enrichment was detected.

### DEGs in response to dehydration treatment

**Fig. 3:**
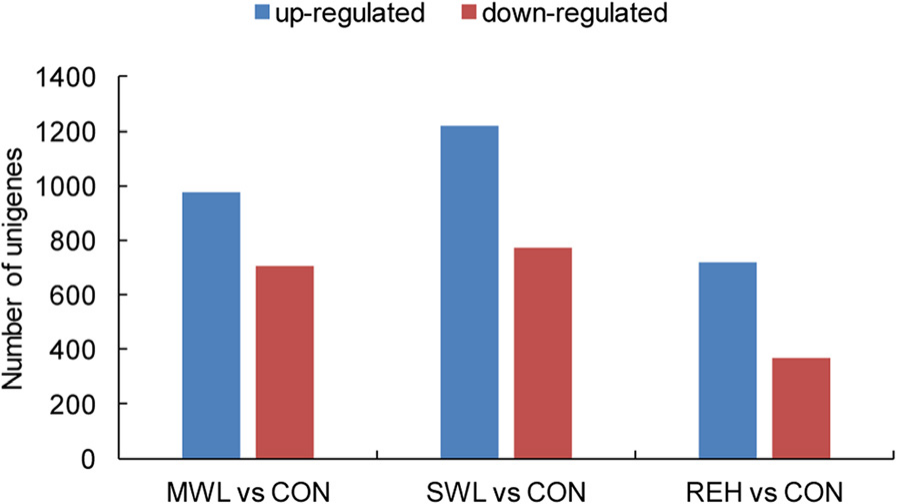
The changes in gene expression profile among the different treatments. Unigenes upregulated (blue) and downregulated (red) in MWL *vs*. CON, SWL *vs*. CON, and REH *vs*. CON were quantified

A total of 1,681 unigenes were significantly (adjusted *P*-value of < 0.05) differentially expressed between CON and MWL. Among these, 977 genes were significantly upregulated, whereas 704 genes were significantly downregulated (Fig. 3, Additional file 5: Table S3). And 1,993 DEGs were found between CON and SWL, which included 1,219 upregulated unigenes and 774 downregulated unigenes (Fig. 3, Additional file 6: Table S4). With regard to DEGs between CON and MWL, GO enrichment tests showed that there were 39 and 19 significantly enriched GO terms in up- and downregulated unigenes, respectively (Additional file 7: Table S5). Similarly, between CON and SWL, there were 33 significantly enriched GO terms in upregulated unigenes (Additional file 8: Table S6 A). Interestingly, within the upregulated GO term associated with cell death, three unigenes (comp20313_c1, comp92397_c0, and comp19022_c0) functioned in the induction of apoptosis and regulation of apoptotic process under moderate dehydration stress (Additional file 5: Table S3). The enriched GO terms that were downregulated in MWL were mainly involved in tRNA aminoacylation and reproductive structure development. However, in contrast to that observed in MWL *vs*. CON, the downregulated unigenes in SWL were significantly enriched in 8 GO terms, which included DNA metabolic process, regulation of mitotic cell cycle, DNA-dependent DNA replication, and DNA repair (Additional file 8: Table S6 B).

Based on KEGG enrichment analysis, the upregulated unigenes in MWL were highly enriched in 7 pathways, including carbon fixation in photosynthetic organisms (PATHWAY: ko00710), pentose phosphate pathway (PATHWAY: ko00030), and biosynthesis of secondary metabolites (PATHWAY: ko01110) (Additional file 9: Table S7 A). Two categories of upregulated unigenes involved in the pathway of carbon fixation in photosynthetic organisms were found, which included C4-pathway and C3-pathway enzymes. Among these upregulated unigenes were 4 unigenes encoding C4-pathway enzymes, which included alanine transaminase (ALT), pyruvate kinase, and aspartate aminotransferase (AST), whereas 18 unigenes encoding C3-pathway enzymes were identified (Table 3). Furthermore, within the biosynthesis of secondary metabolites, 2 upregulated unigenes (comp7250_c0 and comp17143_c0) were found to be involved in trehalose biosynthesis (glucose-1-P → trehalose), which included starch phosphorylase and 1,4-alpha-glucan branching enzyme. In addition, the downregulated unigenes were highly enriched in 8 pathways such as DNA replication (PATHWAY: ko03030) and porphyrin and chlorophyll metabolism (PATHWAY: ko00860) (Additional file 9: Table S7 B). With respect to DEGs between CON and SWL, they were highly enriched in 11 pathways (Additional file 10: Table S8), nearly in accordance with that observed in MWL *vs*. CON. As noted in the heatmap of gene expression, SWL showed an expression pattern that was similar to that observed in MWL.

**Table 3:** Expression variation of unigenes involved in the carbon fixation pathway based on KO database annotation

KO description	Unigene ID	Fold ratio (MWL*/*CON)	Fold ratio (SWL/CON)	Fold ratio (REH*/*CON)
C4 pathway				
Alanine transaminase	comp17488_c0	4.6155 (+)	3.6601 (+)	1.5332
Pyruvate kinase	comp7242_c0	5.2379 (+)	3.1205 (+)	1.2953
	comp7349_c0	-3.8143 (-)	-1.4483	-1.3880
Aspartate aminotransferase	comp17944_c0	10.790 (+)	6.0394 (+)	2.7419
	comp17944_c1	6.3935 (+)	3.8670 (+)	2.3445
Malate dehydrogenase (decarboxylating) (NAD +)	comp15628_c0	3.5910	2.6086 (+)	-1.2936
C3 pathway				
Phosphoribulokinase	comp19110_c0	186.94 (+)	180.14 (+)	16.370 (+)
Phosphoglycerate kinase	comp7447_c0	71.136 (+)	36.733 (+)	11.911 (+)
	comp19092_c0	1230.5 (+)	1212.7 (+)	151.11 (+)
Glyceraldehyde-3-phosphate dehydrogenase (NADP+)	comp15165_c0	326.49 (+)	328.01 (+)	42.775 (+)
Triosephosphate isomerase (TIM)	comp15251_c0	38.314 (+)	28.637 (+)	8.4790 (+)
	comp7503_c1	292.10 (+)	217.79 (+)	47.895 (+)
	comp7503_c0	208.44 (+)	150.65 (+)	36.866 (+)
Fructose-bisphosphate aldolase	comp18097_c0	100.59 (+)	117.82 (+)	9.8847 (+)
	comp7456_c0	9.3775 (+)	6.2615 (+)	2.7501 (+)
	comp17370_c0	-3.7428 (-)	-3.1323 (-)	-1.1993
Fructose-1,6-bisphosphatase I	comp8430_c0	326.94 (+)	212.64 (+)	39.057 (+)
	comp15093_c1	61.987 (+)	73.303 (+)	6.7062 (+)
	comp15093_c0	43.568 (+)	48.392 (+)	5.2318 (+)
	comp16681_c0	5.5282 (+)	4.6589	3.3216 (+)
Transketolase	comp17812_c0	658.76 (+)	570.90 (+)	45.718 (+)
	comp17812_c1	414.49 (+)	410.46 (+)	38.264 (+)
Sedoheptulose-bisphosphatase	comp11960_c0	132.77 (+)	147.66 (+)	18.114 (+)
Ribose 5-phosphate isomerase A	comp9522_c0	53.791 (+)	68.665 (+)	11.184 (+)
Ribulose-phosphate 3-epimerase	comp7253_c0	238.82 (+)	176.38 (+)	33.789 (+)

“(+)” indicates significantly upregulated expression; “(-)” indicates significantly downregulated expression

### DEGs in the initial rapid response to rehydration treatment

Comparative analysis showed that there were 1,086 differentially expressed genes (adjusted *P*-value of < 0.05) between REH and CON, of which 720 unigenes were upregulated and 366 unigenes were downregulated (Fig. 3, Additional file 11: Table S9). GO enrichment testing showed that 28 GO terms, mainly including carbohydrate metabolism (such as carbon utilization, fructose metabolic process, and glycolysis), were significantly enriched among the upregulated genes, whereas no GO terms were enriched among the downregulated unigenes (Additional file 12: Table S10).

Based on KEGG analysis, the upregulated unigenes were highly enriched in carbon fixation in photosynthesis (PATHWAY: ko00710), fructose and mannose metabolism (PATHWAY: ko00051), pentose phosphate pathway (PATHWAY: ko00030), and protein processing in endoplasmic reticulum (PATHWAY: ko04141). Interestingly, within the carbon fixation pathway, only 18 unigenes putatively encoding C3-pathway enzymes were significantly upregulated in response to rehydration treatment, whereas none of genes encoding C4-pathway enzymes were detected (Table 3). Nevertheless, the downregulated unigenes were highly enriched in DNA replication (PATHWAY: ko03030), mismatch repair (PATHWAY: ko03430), nucleotide excision repair (PATHWAY: ko03420), ABC transporters (PATHWAY: ko02010), cell cycle (PATHWAY: ko04110), and base excision repair (PATHWAY: ko03410). Herein, there were 5 downregulated unigenes that were putatively encoding ABCB1, ABCB4, ABCG2, and mitochondrial ABC transporter ATM within the ABC transporters (Additional file 13: Table S11).

### Comparison of DEGs responding to dehydration and rehydration treatments

**Fig. 4:**
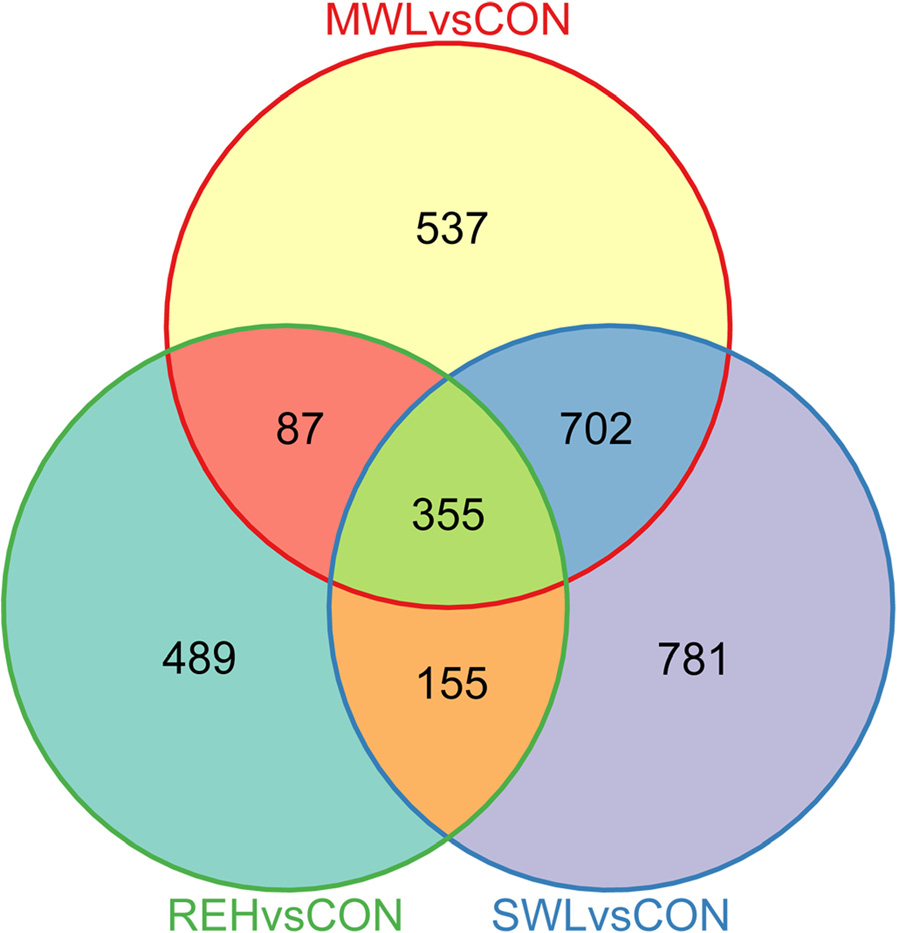
Venn diagram of differentially expressed genes. The sum of the numbers in each large circle represents the total number of differentially expressed genes among various combinations, the overlapping part of the circles represents common DEGs between combinations

The Venn diagram provides an illustration of the overlaps among differentially expressed genes in response to moderate dehydration, severe dehydration, and rehydration (Fig. 4). As shown in Fig. 4, a total of 355 differentially expressed genes were collectively involved in the response to moderate dehydration, severe dehydration, and rehydration. Of these DEGs, 7 unigenes encoding variety of fatty acid desaturases and fatty acid elongase that were involved in biosynthesis of unsaturated fatty acids (UFAs) were identified (Table 4). Meanwhile, 4 unigenes that encoded molecular chaperones, including HSPA4, endoplasmin, HSP70-2, and HSP81-1 were detected. In addition, 6 unigenes encoding various transcription factors, including basic leucine zipper (bZIP), Sigma-70 region 2/PDZ domain, MerR family regulatory protein, and zinc-finger protein, were collectively upregulated or downregulated in three pairwise comparisons (MWL *vs*. CON, SWL *vs*. CON, and REH *vs*. CON) (Table 5).

**Table 4:** Expression variation of unigenes involved in the biosynthesis of UFAs based on Nr and Swiss-Prot database annotations

Gene	Unigene ID	Fold ratio (MWL*/*CON)	Fold ratio (SWL*/*CON)	Fold ratio (REH/CON)
Omega-3 fatty acid desaturase	comp10118_c0	131.72 (+)	139.35 (+)	43.823 (+)
Omega-6 fatty acid desaturase	comp19838_c0	8.9315 (+)	5.9798 (+)	2.7604 (+)
Delta-4 fatty acid desaturase	comp8174_c0	13.267 (+)	11.146 (+)	8.6764 (+)
Delta-5 fatty acid desaturase	comp15504_c0	173.55 (+)	130.32 (+)	20.262 (+)
Delta6-fatty acid desaturase	comp15419_c0	107.65 (+)	70.185 (+)	21.271 (+)
Stearic acid desaturase	comp19376_c0	103.80 (+)	59.047 (+)	18.341 (+)
Delta-6 fatty acid elongase	comp21618_c0	110.24 (+)	242.01 (+)	27.937 (+)

“(+)” indicates significantly upregulated expression

**Table 5:** Change in expression of unigenes encoding transcription factors based on Pfam database annotation

Pfam description	Unigene ID	Fold ratio (MWL*/*CON)	Fold ratio (SWL*/*CON)	Fold ratio (REH/CON)
Sigma-70 region 2/PDZ domain	comp19121_c0	10.253 (+)	6.0771 (+)	2.4975 (+)
MerR family regulatory protein	comp14862_c0	17.136 (+)	19.208 (+)	2.9717 (+)
Zinc finger, C2H2 type	comp15972_c0	21.736 (+)	20.636 (+)	9.1286 (+)
bZIP	comp15892_c0	358.24 (+)	183.78 (+)	40.496 (+)
	comp18331_c0	-3.1832 (-)	-2.6807 (-)	-3.1844 (-)
	comp15544_c1	4.3884 (+)	3.5527 (+)	2.7953 (+)
	comp13101_c0	26.959	27.171 (+)	2.7079
	comp7214_c0	5.1910	6.1292 (+)	2.7562
	comp93507_c0	28.770	61.996 (+)	1.4304
	comp11150_c0	-20.696 (-)	-5.2427	-3.0823
	comp18079_c0	-16.651 (-)	-4.8885	-4.9664
	comp11771_c0	1.4930	1.6420	-2.7064 (-)
	comp12977_c0	1.2026	1.5545	2.0757 (+)
AP2 domain	comp11814_c0	-2.5254	-3.0625 (-)	1.0713
Homeobox-associated leucine zipper	comp23316_c0	2.0326	2.6365	3.4000 (+)
	comp15332_c0	4.4661	3.8530 (+)	-1.0889
	comp17307_c0	3.3889 (+)	1.7018	-1.0460
CCAAT-binding transcription factor (CBF-B/NF-YA) subunit B	comp17988_c0	1.7127	2.7391 (+)	2.2202
Sigma-70 region 3	comp16574_c0	4.4540	2.8341(+)	2.4441 (+)
Sigma-70 region 2	comp18383_c0	-2.8631(-)	-1.7289	-1.3105

“(+)” indicates significantly upregulated expression; “(-)” indicates significantly downregulated expression

Additionally, 537 and 781 specific unigenes were only differentially regulated between MWL and CON, and SWL and CON, respectively. Moreover, a total of 489 specific unigenes showed significantly altered expression during rehydration. Most of these specific unigenes encoded a range of transporters such as aquaporins (comp15218_c0 and comp18064_c0), Na^+^/K^+^ ATPase alpha-subunit 1B (comp16722_c0), ZIP zinc transporters (comp14925_c0 and comp8630_c0), as well as ABC transporters (comp16736_c0, comp16736_c2, comp8118_c0, and comp21051_c0) (Additional file 13: Table S11). In addition, 27 unigenes encoding various ribosomal proteins were also detected, which were involved in ribosome biogenesis/translation, thus strongly suggesting a role for *de novo* translation during rehydration in *Py. haitanensis*.

### Features of the gene encoding 1,4-alpha-glucan branching enzyme

With respect to the unigene (cDNA sequence) encoding 1,4-alpha-glucan branching enzyme, which was identified as *Ph*SBE, it had 4,140 nucleotide residues in which was contained an 2,268 bp open reading frame, which was predicted to code for 755 amino acids with a molecular weight of 85.2 kDa (Additional file 14: Figure S3 A). Further alignment analysis revealed the gene was interrupted by the 291 bp and 208 bp introns, indicating that three exons and two introns were contained in the gene (Additional file 14: Figure S3 B). Analysis of amino acid sequence showed that it matched with Alpha-amylase_C domain, without signal peptide. Multiple sequence alignments showed that *Ph*SBE was highly conserved with 1,4-alpha-glucan branching enzyme (starch branching enzyme) found in other species. It identified 70 % with *Chondrus crispus* (XP_005716136.1), 69 % with *Gracilaria gracilis* (AAB97471.1), 67 % with *Cyanidioschyzon merolae* strain 10D (XP_005536101.1), 59 % with *Galdieria sulphuraria* (XP_005703889.1), 56 % with *Arabidopsis thaliana* (NP_195985.3), 57 % with *Zea mays* (NP_001105316.1) and 56 % with *Oryza sativa* (BAA82828.1) (Additional file 15: Figure S4).

### Validation of RNA-Seq-based gene expression

**Fig. 5:**
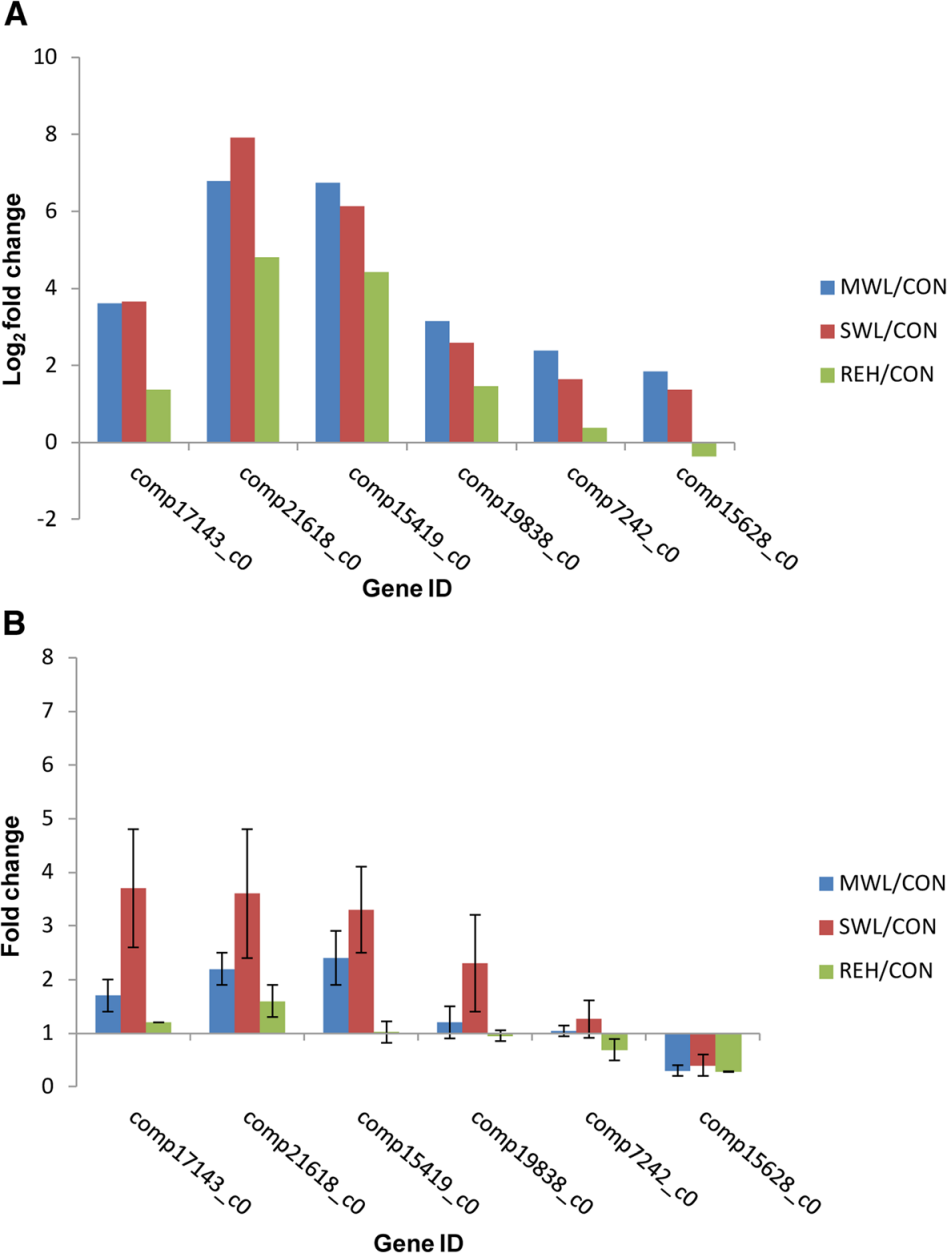
qRT-PCR analysis of 6 randomly selected unigenes. **a** Gene expression data for DGE analysis. The relative expression changes of the genes were calculated as the log2 vaule of MWL/CON, SWL/CON and REH/CON and shown on the y-axis. **b** The qRT-PCR analysis of gene expression data. Expression ratios of these genes in MWL, SWL and REH were compared to CON, respectively. Error bars represent the SD for three biological replicates

To validate the expression profiles obtained by RNA-Seq, qRT-PCR was performed on six genes selected at random with high or low expression levels. Expression comparisons were performed between MWL and CON, SWL and CON, and REH and CON by qRT-PCR. Generally, the expression profiles of the genes assayed show upregulated expression in response to MWL and SWL, and marginal increases in response to REH, nearly confirming the DGE expression data (Fig. 5). Two genes encoding omega-6 fatty acid desaturase (comp19838_c0) and pyruvate kinase (comp7242_c0) however showed downregulated expression in response to REH, which showed marginal upregulated expression in response to REH in DGE expression data. The relative expression of the gene encoding malate dehydrogenase (comp15628_c0) was inverse to the expected expression, with downregulated expression in response to MWL and SWL.

## Discussion

### The mechanism of desiccation tolerance in *Py. haitanensis*

Carbon fixation is the most important biological process in all photosynthetic organisms, which can be divided into three general categories: C3, C4, and Crassulacean acid metabolism (CAM) [31]. The Calvin cycle (C3 pathway) is the most basic and universal form of net carbon fixation in plants, algae, and cyanobacteria. However, the C4 pathway is an adjunct of the C3 pathway that developed novel and efficient CO_2_ concentration mechanisms to enhance ribulose bisphosphate carboxylase/oxygenase (Rubisco) performance even at limiting ambient CO_2_ levels [32]. The C4 pathway has long been studied in higher plants with much higher photosynthetic rates than C3 plants [32, 33]. Nevertheless, the C4 pathway in algae has also been the subject of several reports in the past decade. The C4 pathway is important in carbon accumulation and photosynthetic carbon fixation in the marine diatom *Thalassiosira weissflogii* at conditions of low (atmospheric) CO_2_ [34]. By genome analysis, all the required genes involved in C4 photosynthesis were presented in unicellular green algae *Ostreococcus tauri*, which is the smallest free-living eukaryote yet described [35]. With respect to the red algae, Yang et al. [19] reported that, except for pyruvate phosphate dikinase, almost all genes involved in the C4 pathway have been identified in *Py. yezoensis*. Meanwhile, *C. crispus* genome possesses two NADP-malic enzymes (ME), three malate dehydrogenases (MDHs), and one phosphoenolpyruvate carboxylase (PEPC), and thus a malate-based C4-like carbon fixation pathway was considered to be present in the algal species [36]. In *Py. haitanensis*, the expression level of Rubisco (key gene in the C3 pathway) was significantly lower in the conchocelis than that in the thallus, whereas the expression levels of PEPC and phosphoenolpyruvate carboxykinase (PEPCK), which are the key genes in the C4 pathway, were significantly higher in the conchocelis than that in the thallus [22]. The C4-like pathway was thus considered to play an important role in the fixation of inorganic carbon in the conchocelis stage of *Py. haitanensis* [22]. Interestingly, by DGE analysis, a large number of unigenes encoding C4 and C3 pathway-related enzymes were significantly upregulated in response to both MWL and SWL treatment, except for 2 unigenes (comp7349_c0 and comp17370_c0). Therefore, C4 pathway- and C3 pathway-related genes may collectively play an important role in the fixation of inorganic carbon when *Py. haitanensis* gametophytes were subjected to desiccation stress.

In most cases, the ability of the plant to survive desiccation correlates with the accumulation of carbohydrates. Trehalose (α-D-glucopyranosyl-(1,1)-α-D-glucopyranoside) is a non-reducing disaccharide of two glucose units that is predominantly present in desiccation-tolerant lower organisms, including some vascular plants such as *Selaginella tamariscina*, the moss, *Tortula ruralis*, and algae [37–39], and functions as a stress protection metabolite in the stabilization of biological structures under various abiotic stresses [40]. In the present study, two upregulated unigenes encoding starch phosphorylase and 1,4-alpha-glucan branching enzyme were detected in dehydration treatment, which were predicted to involved in trehalose biosynthesis (glucose-1-P → trehalose). The two genes were also reported to be present in *C. crispus* genome, as well as *G. gracilis*, *C. merolae*, and *G. sulphuraria* genomes [36, 41–43]. In bacteria, fungi, plants and invertebrates, UDP-glucose is a precursor for making trehalose. The production of UDP-glucose from glucose 1-phosphate can be mediated by a classic UTP-glucose-1-phosphate uridylyltransferase (UGP2, EC 2.7.7.9) [44, 45]. And the release of the glucose-1-P (G1P) from the non-reducing ends of the outer chains of polysaccharides composed of a-1,4-linked glucose residues, such as amylopectin, is catalysed by starch phosphorylase. Furthermore, 1,4-alpha-glucan branching enzyme catalyses the biosynthesis of amylopectin from amylose. It is supposed that two upregulated unigenes may promote the release of the glucose-1-P (G1P) by degradation of amylopectin-like floridean starch, which further promote accumulation of trehalose, contributing to protecting the algae against osmotic stress in intertidal environment.

Chlorophyll biosynthesis in plants is subjected to modulation by various environmental factors. For example, in etiolated rice seedlings, ionic imbalance due to salinity stress resulted in additional downregulation (41 %–45 %) of seedling dry weight, as well as chlorophyll and carotenoid levels [46]. Meanwhile, in *Helianthus annuus* L. plants, salt stress more drastically affects chlorophyll synthesis (decreasing α-linolenic acid synthesis) than chlorophyllase-mediated degradation [47]. In the current study, 7 down-regulated unigenes encoding chlorophyll biosynthetic pathway enzymes in MWL, i.e. oxygen-dependent protoporphyrinogen oxidase and magnesium chelatase subunit H were detected, including one downregulated unigene that encoded for glutamate-1-semialdehyde 2,1-aminomutase in SWL. The downregulation of these enzymes resulted in a reduction in chlorophyll synthesis, which was similar to that observed in higher plants. Previous reports have shown that photosynthetic CO_2_ fixation in higher plants is inhibited by water-deficit stress, and the excitation energy by chlorophyll can greatly exceed the demand of the Calvin cycle for ATP and NADPH, thus resulting in the overreduction of the electron transport chain and enhanced generation of ROS, ultimately leading to inhibition of PS II reaction centers, damage to the ATP synthesizing machinery, and a decrease in photosynthetic rate [48–51]. Based on these findings, it is therefore assumed that in *Py. haitanensis*, the downregulation of chlorophyll biosynthesis contributes to the inhibition of the accumulation of highly photosensitive photodynamic tetrapyrroles that generates ROS under stress conditions. Thus, the downregulation of chlorophyll biosynthesis is advantageous in *Py. haitanensis* because the accumulation of toxic ROS is reduced.

Apoptosis is a basic biological process that functions in various aspects of animal and plant development and in their responses to stress [52, 53]. Cysteine proteases (caspases) are key components of animal apoptosis [54], and the activation of cysteine proteases constitutes the critical point in the apoptosis pathway of animal cells [55, 56]. However, while animals have caspase genes, plants such as *Arabidopsis* and rice do not harbor orthologous caspase sequences in their genomes, whereas caspase-like activities have also been detected in plant cells [54]. Instead, plant genomes encode several related proteins, called metacaspases, which were found to mediate apoptosis [57]. Meanwhile, there is evidence for a mitochondrial release of apoptosis activating molecules following a moderate level 55 °C heat shock [58]. Recently, in red algae *C. crispus*, several homologues of apoptosis- and programmed cell death-related genes were also identified in the genome, including metacaspases, apoptosis inducing factor, Bax inhibitor, etc [36]. Furthermore, in *Py. yezoensis*, the expression of genes encoding metacaspases was found to be upregulated under high-temperature stress, revealing their important roles in *Py. yezoensis* acclimation to heat stress [59]. Similar to higher plants and other red algae, classical caspase genes or transcripts were not found in *Py. haitanensis*. It is worth noting that one unigene (comp18960_c0) encoding metacaspase was also found to be up-regulated under desiccation stress, but none was found in rehydration. And three upregulated unigenes putatively encoding for the mitochondria-derived activator of caspases were detected under desiccation stress, functioning in the induction of apoptosis and the regulation of apoptotic process (Additional file 5: Table S3). It is therefore likely that in response to desiccation stress, the apoptogenic proteins such as mitochondria-derived activator of caspases, were released from the mitochondria, which led to the activation of metacaspase molecules, and thereby resulting in apoptosis. *Pyropia* and other red algal species grown in harsh intertidal habitats posses an nearly overall apoptosis picture similar of the one found in plants.

### The initial rapid response of *Py. haitanensis* to rehydration

Rehydration-induced revival of various metabolic processes in plants requires an efficient transport machinery. Water transport is of great importance to water homeostasis in plants subjected to dehydration and rehydration. Water channel proteins (known as aquaporins) often facilitate osmotic water flow across membranes, and are critical for osmotic regulation and glycerol transport in vascular plants, which in turn can mediate CO_2_ permeability and photosynthetic activity [60, 61]. The expression of several aquaporins is regulated in response to environmental factors such as dehydration stress, salinity stress, and rehydration [62–64]. Two recent studies clearly demonstrated the effective involvement of aquaporins in conferring stress tolerance in higher plants [65, 66]. The aquaporins in algae have also been the subject of several reports in recent decades. Henzler and Steudle [67] demonstrated that algal membranes can be modeled as composite structures in which water is transported through highly selective aquaporins, while solutes permeate via other routes through the membrane. Akai et al. [68] further reported that in the cyanobacterium *Synechocystis* sp. PCC 6803, the aquaporin (AqpZ) was involved in cell volume regulation and functioned as a water transport system that responds to daily oscillations of intracellular osmolarity. The published genome of *Chlamydomonas* contains three putative aquaporins: MIP1 (Cre12.g549300.t1.2), MIP2 (Cre17.g711250.t1.2), and MIP3 (Cre01.g038800.t1.2) [69]. Moreover, MIP1 was found to be localized to the contractile vacuole in *Chlamydomonas*, functioning *in vivo* as a water channel [70]. In the red alga *Porphyra purpurea*, some EST contigs putatively encoding aquaporins have been detected, although information on the specific functions of these channel proteins is currently limited [20]. Similarly, in this work, two aquaporin unigenes were found to have altered expression in response to rehydration, of which one aquaporin unigene was upregulated and the other was downregulated, whereas none was observed in differentially expressed genes between MWL and CON, as well as SWL and CON. Plants aquaporins exhibit a high diversity with many different isoforms. Molecular analyses on regulation of the whole aquaporin family have often revealed complex transcriptional and post-translational response patterns, with sometimes opposite profiles between isoforms [71]. It seems possible that above two unigenes code for different isoforms, and the increase in expression levels of one aquaporin gene might provide *Py. haitanensis* additional ability to increase the water permeability of the cells under rehydration. On the other hand, the *Py. haitanensis* may avoid water loss by downregulating another aquaporin gene during rehydration. Thus, in *Py. haitanensis*, these various aquaporins were actively involved in modulating transmembrane water transport during the initial phases following rehydration when subjected to dehydration stress. Although in evolutionary viewpoint, the genome architectures of red algae are distantly related with the vascular plants [72], the aquaporins could represent the ancient water transport system throughout evolution of plants. Further work is clearly required to completely understand the roles and involvement of the aquaporins in the intertidal algae response to rehydration.

Additionally, solute (osmolytes) transport in and out of the vacuoles of desiccation-tolerant angiosperms occurs during desiccation and rehydration [73]. In addition, there is a significant representation within the transporter subcategory in the rehydration transcriptome of the desiccation-tolerant bryophyte *T. ruralis* [64]. Similarly, several transport-associated unigenes were exclusively detected in response to rehydration in the present study, which encode ABC transporters, ion transporters, phosphate transporters, and aquaporins, thus suggesting that these may constitute the transport machinery of *Py. haitanensis* under rehydration conditions. In particular, the unigenes encoding ABC transporters were downregulated during rehydration (Additional file 13: Table S11). ABC transporters have been recognized to participate in a multitude of physiological processes that allow the plant to adapt to changing environments and cope with biotic and abiotic stresses, as well as detoxification processes [74]. Chan et al. has also identified several ABC transporters that were related to multidrug resistance, bile salt pumps, and the transport of lipids into the plastid in *P. purpurea* and *P. umbilicalis* transcriptome [20]. Therefore, it is likely that during rehydration, the downregulation of unigenes encoding ABC transporters instantly matched the decrease in the requirement for lipids and lipophilic compounds that protect the plant from biotic and abiotic stresses, with the revival of many biological metabolism.

Facultative C3-CAM species such as *Guzmania monostachia* (Bromeliaceae) and *Talinum triangulare* (Portulacaceae) can be induced by various environmental factors such as drought stress and salinity to utilize, and then returned to a typical C3 condition after a subsequent period of seven days of rehydration [75]. Unlike that observed during dehydration treatment, many of the unigenes associated with the C4 pathway for photosynthetic carbon reduction were also not significantly altered, whereas many unigenes in *Py. haitanensis* that were involved in a putative C3 pathway for photosynthetic carbon assimilation were upregulated under rehydration, suggesting the return of a typical C3 condition after a subsequent period of one half hour of rehydration. These results revealed that *Py. haitanensis* employed different carbon-concentrating mechanisms based on the actual environmental factor.

### Genes collectively responding to dehydration and rehydration in *Py. haitanensis*

Unsaturated fatty acids (UFAs) have profound effects on the fluidity and function of biological membranes. Plants, animals, and microorganisms regulate the synthesis of UFAs to remodel membrane fluidity during changing environmental conditions as well as in response to nutrients [76]. Various fatty acid desaturases (FAD) are key enzymes in the synthesis of unsaturated fatty acids. In higher plants, drought tolerance is considered to be closely correlated with the level of unsaturated fatty acids [77–79]. For example, the increase in α-linolenic acid (18:3, LA) levels in *Nicotiana tabacum* cells due to the overexpression of the ω-3 fatty acid desaturases FAD3 and FAD7 enhances tolerance to drought stress [77], whereas non-tolerant plants decline their fraction of 18:3 [78]. In addition, several studies have been performed to explore the role of unsaturated fatty acids in red algae. Sun et al. [59] reported that in *Py. yezoensis* cold stress caused an increase in the expression of FAD to improve the proportion of polyunsaturated fatty acids. Furthermore, the amounts of polyunsaturated fatty acids, especially those of 20:4 and 20:5, were decreased significantly in conchocelis of *Py. haitanensis* with the increase in temperature whereas thallus exhibited only minor differences in fatty acid levels between control and the heat stress treatments with a slight decrease of 20:5 and an increase of 20:4 [80]. In the present study, different from that in response to temperature stress, all of differentially expressed unigenes encoding fatty acid desaturases and fatty acid elongase were collectively upregulated in response to dehydration and rehydration (Table 4). Thus, in *Py. haitanensis*, fatty acid desaturases and fatty acid elongase were involved in the increase in blade membrane unsaturation in response to dehydration and rehydration, all of which contributes to their osmotic acclimation to the intertidal habitat.

Families of transcription factors functioned downstream of signaling cascades that were related to biological and environmental stimuli. Several members of these families were previously identified to be responsive to various stresses [81] such as bZIP to drought and abscisic acid (ABA), MYB to dehydration, zinc finger to cold and drought, bHLH and NAC to drought, salinity, and ABA. Recently, transcription factors such as bZIP and zinc-finger protein were present both in stressed and rehydrated samples of poplar (*Populus alba* L.) [82]. Similarly, of the 20 differentially expressed unigenes encoding transcription factors in MWL, SWL, and REH as compared to CON, 6 unigenes, which included bZIP and zinc-finger protein, were collectively upregulated or downregulated in dehydration/desiccation-stressed and rehydrated samples of *Py. haitanensis* (Table 5). In higher plants, transcription factors belonging to the class of DRE-binding protein (DREB)/C-repeat-binding factor (CBF), bZIP, MYC, and MYB homologs are involved in ABA-dependent or ABA-independent stress signaling pathways [83–85]. Interestingly, in the present study, one unigene encoding 9-cis-epoxycarotenoid dioxygenase (NCED), which is an important enzyme in the synthesis of the phytohormone ABA [86], was found to be upregulated in response to moderate dehydration, severe dehydration, and rehydration, thus revealing endogenous ABA levels may be induced as a result of dehydration and rehydration. Several of the drought-related genes can be induced by ABA. For example, the *Arabidopsis rd22 BP1* gene that encodes a Myc homologue transcription factor has been shown to be induced by dehydration, high-salt conditions, and ABA [83]. In *Py. haitanensis*, the upregulation of several unigenes encoding bZIPs by endogenous ABA in response to dehydration and rehydration still needs further verification. In addition, research studies that examine the roles and involvement of these transcription factors in stress signaling pathways of *Py. haitanensis* are also warranted.

## Conclusions

The combination of *de novo* transcriptome sequencing and DGE analysis based on the NGS technology is a powerful method for identifying candidate genes and key metabolic processes involved in the response to osmotic stress in *Pyripoa* species. Using this method, genes associated with the C4 and C3 pathways, trehalose biosynthesis, porphyrin and chlorophyll metabolism, induction of apoptosis, reproductive structure development, and other carbohydrate metabolic process were determined to be involved in the desiccation response. On the other hand, we demonstrated the mechanisms involved in the initial response to rehydration based on the following perspectives: multiple transport machinery such as aquaporins and ABC transporters, and the return of the normal C3 condition. In addition, the synthesis of unsaturated fatty acids, various transcription factor families, and molecular chaperones have collectively been implicated in the process of dehydration and rehydration in *Py. haitanensis*. The transcriptome data generated in the present study can serve as an important resource for understanding tolerance mechanisms of *Py. haitanensis* as it thrives in its unique intertidal environment.

## Methods

### Algal materials and cultivation conditions

To eliminate the interference caused by genotypic differences, a lab-cultured pure line PH-38 of *Py. haitanensis* was used in the experiments. The sporophytes (conchocelis) of this pure line were directly developed from a single somatic cell that was enzymatically isolated from a farmed thallus and then cultured in bubbling sterilized seawater with Provasoli’s enrichment solution medium (PES) under 20 μmol photons · m^–2^ s^–1^ at 24 ± 1 °C and a 12:12 light:dark (L:D) photoperiod. Gametophytes (thalli) were formed from germination of the conchospores that were released from the mature sporophytes (conchosporangia), which were cultured in running sterilized seawater with PES under 50 μmol photons · m^–2^ s^–1^ at 20 ± 1 °C and a 12:12 light:dark (L:D) photoperiod.

### Experimental design and sampling

To simulate conditions in the open sea, the gametophytes were allowed to acclimate for 2 weeks in running sterilized seawater (at 20 ± 1 °C, 12:12 light:dark (L:D) photoperiod, and 1,250 μmol photons · m^-2^ s^-1^) prior to performing the experiments. Following acclimation, the algae were exposed to various abiotic stress treatments (for whole transcriptome sequencing) and dehydration/rehydration treatments (for DGE sequencing).

Due to limited *Py. haitanensis* genome information, we first generated a global transcriptome for this species. The materials included samples from different developmental phases and treatments (Additional file 1: Table S1). After harvesting and weighing, the samples were immediately frozen in liquid nitrogen.

For DGE analysis, the gametophytes were subjected to dehydration and rehydration by exposing to air and transferring these back to seawater. The algal samples in normal conditions (CON) were harvested before the dehydration treatments. The algal samples of moderate water loss (MWL) were collected when the algae reached a water loss level of 30 ± 5 %, whereas the algal samples of severe water loss (SWL) were collected when the algae reached a water loss level of 80 ± 5 %. For rehydration (REH), severe dehydrated algae were transferred back to normal conditions and a representative sample was collected after 0.5 h. All treatments were performed at 20 ± 1 °C and 1,250 μmol photons · m^-2^ s^-1^. Two independent replicates were used for each treatment. Water loss was determined according to Kim et al. [87]. Algal samples from each treatment were bulked separately prior to RNA isolation. All samples for stress treatments were immediately collected and frozen in liquid nitrogen.

### RNA isolation

Total RNAs were extracted from each sample using E.Z.N.A.™ Plant RNA Kit (Omega Bio-Tek, Norcross, GA, USA), and contaminating DNA was digested with RNase-Free DNase I (TIANGEN, Beijing, China), according to the manufacturer’s instructions. The status of RNA degradation and contamination were monitored on 1 % agarose gels. RNA purity was checked using a NanoPhotometer spectrophotometer (IMPLEN, CA, USA). RNA concentration was measured using a Qubit RNA Assay Kit and a Qubit 2.0 Fluorometer (Life Technologies, CA, USA). RNA integrity was assessed using the RNA Nano 6000 Assay Kit of the Bioanalyzer 2100 system (Agilent Technologies, CA, USA). To obtain a comprehensive list of transcripts used in transcriptome sequencing, equal amounts of total RNA from samples of different developmental phases and treatments were pooled together.

### Library preparation for transcriptome and DGE sequencing

For transcriptome and DGE sequencing, a total of 6 μg of RNA per sample was used as input material. All nine RNA samples (including a pooled RNA sample for *de novo* transcriptome sequencing and eight RNA samples for DGE sequencing) had RIN values > 7.0. Sequencing libraries were generated using an Illumina TruSeq RNA Sample Preparation Kit (Illumina, San Diego, USA) following the manufacturer’s recommendations, and nine index codes were added to attribute sequences to each sample. Briefly, mRNA was purified from total RNA using oligo (dT) magnetic beads. Following purification, mRNA was fragmented into smaller pieces using divalent cations under elevated temperature in an Illumina proprietary fragmentation buffer. The cleaved RNA fragments were used for first-strand cDNA synthesis using random oligonucleotides and SuperScript II. Second-strand cDNA synthesis was subsequently performed using DNA polymerase I and RNase H. Remaining overhangs were converted into blunt ends via exonuclease/polymerase activities and then the enzymes were removed. After adenylation of the 3' ends of DNA fragments, Illumina PE adapter oligonucleotides were ligated to prepare for hybridization. To select cDNA fragments of preferentially 200 bp in length, the library fragments were purified using the AMPure XP system (Beckman Coulter, Beverly, USA). DNA fragments with ligated adaptor molecules on both ends were selectively enriched using Illumina PCR Primer Cocktail in a 10-cycle PCR reaction. Products were purified (AMPure XP system) and quantified using the Agilent high-sensitivity DNA assay on the Agilent Bioanalyzer 2100 system. Clustering of the index-coded samples was performed on a cBot Cluster Generation System using the TruSeq PE Cluster Kit v3-cBot-HS (Illumina, San Diego, USA), according to the manufacturer’s instructions. After cluster generation, the library preparations were sequenced on an Illumina Hiseq 2000 platform. Together, the library from the pooled RNA sample was sequenced with the 100-bp pair-end reads for *de novo* transcriptome analysis. The other eight libraries were sequenced with the 100-bp single-end reads for DGE analysis.

### Quality control

Raw data (raw reads) in fastq format were first processed using in-house Perl scripts. In this step, clean data (clean reads) were obtained by removing reads containing adapter sequences, reads containing ploy-N, and low quality reads from the raw data. Simultaneously, the Q20, Q30, GC content, and sequence duplication level of the clean data were calculated. All downstream analyses were therefore based on clean, high-quality data.

### *De novo* assembly and annotation

After quality filtering, transcriptome *de novo* assembly was performed using the short reads assembling program, Trinity [28], with min_kmer_cov set to 2 and all other parameters set to default. The optimal assembly results were chosen according to the assembly evaluation. Clustering analysis was then performed to generate a unigene database that comprised potential alternative splicing transcripts.

The unigenes were compared against the NCBI Nr and Nt databases, as well as Swiss-Prot database using BLAST 2.2.27+, with an *E*-value of 1e-10, 1e-5, and 1e-5, respectively. Gene names were assigned to each unigene based on the best BLAST hit (highest score). The unigene sequences were also aligned to the KOG database to predict and classify functions using BLAST 2.2.27+, with an *E*-value of 1e-3. The unigene sequences were searched against the Pfam database to predict functional domain and protein family using Hmmerscan (HMMER 3.0 package), using an inclusion *E*-value of 0.01.

To annotate the unigenes with GO terms that described biological processes, molecular functions, and cellular components, the Nr and Pfam annotation results were imported into the Blast2GO program [88], a software package that retrieves GO terms and subsequently identifies and compares gene functions.

To obtain an overview of gene pathways networks, KEGG pathways were assigned to the unigenes using the online KEGG Automatic Annotation Server (KAAS; http://www.genome.jp/kegg/kaas/). The bi-directional best hit (BBH) method was used to obtain

KEGG Orthology (KO) assignment [89]. The output of KEGG analysis included KO assignments and KEGG pathways that were populated with the KO assignments.

### Reads mapping to the reference genome

Prior to mapping reads to the reference database, raw reads were transformed into clean reads as earlier described. The *de novo* transcriptome database was selected as reference genome. The clean reads of the samples were mapped to the reference genome, and obtained from the annotation information of each sample using the RSEM (v1.2.0) software package [29].

### Quantification of gene expression level

HTSeq software (www-huber.embl.de/users/anders/HTSeq/) was used to determine the number of reads that mapped to each gene. To compare expression abundance among samples, read counts were normalized to the reads per kilobase of exon model per million mapped reads (RPKM) [90] to obtain the relative levels of expression. An RPKM value of > 0.3 was defined as the threshold of significant gene expression.

### Differential expression analysis

Differential expression analysis was performed using DESeq packages [30] for comparisons among samples with two biological replicates. Transcript abundances for each gene were expressed as the weighted mean of read counts from two replicates normalized to the overall library size (also known as ‘base mean’). The relationship between the relative abundance of each gene in parallel libraries was statistically assessed using Pearson’s correlation coefficient. *P*-values (adjusted for false discovery rate) were generated for each gene in pair-wise comparisons among samples. An adjusted *P*-value of 0.05 was set as the threshold for significant differential expression. Variance stabilized data obtained with DESeq was used to generate the heatmaps of differentially expressed genes.

### GO and KEGG enrichment analysis of differentially expressed genes

To study the biological significance of differentially expressed genes, gene ontology (GO)-based enrichment tests were conducted using GOseq [91]. GO terms with corrected *P*-values < 0.05 were considered significantly enriched by differentially expressed genes.

We used the KOBAS software [92] to test the statistical enrichment of differentially expressed genes in KEGG pathways. Pathways with corrected *P*-values < 0.05 were considered significantly enriched by differentially expressed genes.

### Sequence analysis of the functional gene

The unigene encoding 1,4-alpha-glucan branching enzyme (*Ph*SBE) was selected and further analyzed using the BLAST and ORF-finder at NCBI (http://www.ncbi.nlm.nih.gov/) for the homology study and putative ORF prediction. In order to identify the intron, alignment of the ORF and the gene sequence from draft genome (unpublished data) was performed using Spidey at NCBI. The analysis of deduced protein was carried out using ExPASy tool (http://www.expasy.org), which has hyperlinks to different prediction programs, including SingnalP for single peptide prediction. The domain prediction of *Ph*SBE was assessed using the Pfam hidden Markov model (HMM) algorithm (http://pfam.xfam.org/search). The DNAMAN program was used for multiple sequence analysis.

### Quantitative real-time PCR (qRT-PCR) validation

Total RNA was extracted as described for DGE library preparation and sequencing. For the first-strand cDNA synthesis experiment, a Transcriptor First Stand cDNA Synthesis Kit (Roche) was used following the manufacturer’s instructions. The expression levels of selected genes were analyzed by qRT-PCR with a Light-Cycle® 480 Real-Time PCR System. The reactions were performed in 20 μL volumes containing 10 μL of 2 × SYBR® Premix Ex Taq (TaKaRa Biotech Co., Dalian, China), 0.6 μL of each primer (0.3 μM final concentration of each primer), 2 μL of the diluted cDNA mix, and 6.8 μL of RNA-free water. The thermal profile for qRT-PCR was 95 °C for 30s, followed by 40 cycles of 95 °C for 5 s, 57 °C for 30s and 72 °C for 30s. Melting curves for each amplicon were then analyzed to verify the specificity of each amplification reaction. No-template controls were included for each primer pair and each PCR reaction was carried out in three biological replicates. The sequences of the primers used are given in Additional file 16: Table S12. The ubiquitin conjugating enzyme (UBC) and 18 s ribosomal RNA (18 s) genes were used as internal controls [80, 93]. The 2^-ΔΔCt^ method was used to calculate relative gene expression values [94].

### Availability of supporting data

The data sets supporting the results of this article are available in the Sequence Read Archive (SRA) database, accessible through NCBI BioProject ID PRJNA282903 for the transcriptome data (http://www.ncbi.nlm.nih.gov/bioproject/PRJNA282903) and PRJNA283027 for the DGE data (http://www.ncbi.nlm.nih.gov/bioproject/PRJNA283027).

## Additional files

Additional file 1: Table S1. Treatments and developmental stages for transcriptome sampling and summary of the *Py. haitanensis* transcriptome. (DOCX 17 kb) Additional file 2: Figure S1. Log-log plot showing the dependence of unigene lengths on the number of reads assembled into each unigene. (PDF 1390 kb) Additional file 3: Table S2. Summary of clean reads aligned uniquely to the reference transcriptome for each library. (DOCX 13 kb) Additional file 4: Figure S2. Scatterplot showing the correlation of expression levels between two replicates. (PDF 2576 kb) Additional file 5: Table S3. (A) The comparison of expressed gene lists between MWL and CON. (B) Differentially expressed genes between MWL and CON. Readcount: transcript abundances for each gene. Pval: *P* value. Padj: adjusted *P*-value. Adjusted *P*-value of < 0.05 were used as the threshold to judge the significance of gene expression difference. (XLSX 2517 kb) Additional file 6: Table S4. (A) The comparison of expressed gene lists between SWL and CON. (B) Differentially expressed genes between SWL and CON. Readcount: transcript abundances for each gene. Pval: *P* value. Padj: adjusted *P*-value. Adjusted *P*-value of < 0.05 were used as the threshold to judge the significance of gene expression difference. (XLSX 2517 kb) Additional file 7: Table S5. (A) GO enrichment analysis of the upregulated genes in MWL compared to CON. (B) GO enrichment analysis of the downregulated genes in MWL compared to CON. (XLSX 436 kb) Additional file 8: Table S6. (A) GO enrichment analysis of the upregulated genes in SWL compared to CON. (B) GO enrichment analysis of the downregulated genes in SWL compared to CON. (XLSX 429 kb) Additional file 9: Table S7. (A) KEGG pathway enrichment analysis of the upregulated genes in MWL compared to CON. (B) KEGG pathway enrichment analysis of the downregulated genes in MWL compared to CON. (XLSX 33 kb) Additional file 10: Table S8. KEGG pathway enrichment analysis of the differentially expressed genes in SWL compared to CON. (XLSX 26 kb) Additional file 11: Table S9. (A) The comparison of expressed gene lists between REH and CON. (B) Differentially expressed genes between REH and CON. Readcount: transcript abundances for each gene. Pval: *P* value. Padj: adjusted *P*-value. Adjusted *P*-value of < 0.05 were used as the threshold to judge the significance of gene expression difference. (XLSX 2323 kb) Additional file 12: Table S10. (A) GO enrichment analysis of the upregulated genes in REH compared to CON. (B) GO enrichment analysis of the downregulated genes in REH compared to CON. (XLSX 334 kb) Additional file 13: Table S11. Differentially expressed genes encoding ABC transporters between REH and CON based on KO database annotation. (DOCX 15 kb) Additional file 14: Figure S3. (A) Nucleotide and deduced amino acid sequence of cDNA encoding 1,4-alpha-glucan branching enzyme (*Ph*SBE). Nucleotide residues are numbered in the 5’ to 3’ direction. Amino acid residues are numbered in the N- to C-terminal direction. The start codon is marked in red color font. An asterick denotes the stop codon. (B) Genomic sequence structure of the *Ph*SBE ORF (5’ to 3’ direction). 1 (arrow) represents start site of translation, 2767 represents end site of translation. Exon 1 (1-28), exon 2 (320-401) and exon 3 (610-2767) are depicted as red boxes. Intron 1 (29-319) and intron 2 (402-609) are depicted as narrow blue boxes. (PDF 170 kb) Additional file 15: Figure S4. Multiple sequence alignment of 1,4-alpha-glucan branching enzymes from different species. 1,4-alpha-glucan branching enzymes were chosen from different species and aligned by DNAMAN. Sequence in frame means Alpha-amylase_C domain. The accession numbers of these sequences were showed as follows: *Chondrus crispus* (XP_005716136.1), *Gracilaria gracilis* (AAB97471.1), *Cyanidioschyzon merolae* strain 10D (XP_005536101.1), *Galdieria sulphuraria* (XP_005703889.1), *Arabidopsis thaliana* (NP_195985.3), *Zea mays* (NP_001105316.1) and *Oryza sativa* (BAA82828.1). (PDF 12907 kb) Additional file 16: Table S12. Primers used in qRT-PCR for validating differentially expressed genes. (DOCX 14 kb)

## Abbreviations

DGE: Digital gene expression profile
Nr: NCBI non-redundant protein
Nt: NCBI non-redundant nucleotide sequence
Swiss-Prot: The manually annotated and curated protein sequence database
Pfam: Protein family
KOG: euKaryotic Ortholog Groups
GO: Gene ontology
KEGG: Kyoto Encyclopedia of Genes and Genomes
KO: KEGG Orthology
CON: Normal conditions
MWL: Moderate water loss
SWL: Severe water loss
DEGs: Differentially expressed genes
REH: Rehydration
ALT: Alanine transaminase
AST: Aspartate aminotransferase
PEPC: Phosphoenolpyruvate carboxylase
PEPCK: Phosphoenolpyruvate carboxykinase
ME: NADP-malic enzyme
MDH: Malate dehydrogenase
Rubisco: Ribulose 1,5-bisphosphate carboxylase-oxygenase
UFAs: Unsaturated fatty acids
LA: α-Linolenic acid
FAD: Fatty acid desaturases
ABA: Abscisic acid
bZIP: Basic leucine zipper
DREB: DRE-binding protein
CBF: C-repeat-binding factor
NCED: 9-Cis-epoxycarotenoid dioxygenase
RPKM: Reads per kilobase of exon model per million mapped reads
UBC: Ubiquitin conjugating enzyme
18 s: 18 s ribosomal RNA
