# Supplementary material for: Genome-wide expression profiles of Pyropia haitanensis in response to osmotic stress by using deep sequencing technology
Source: BMC Genomics. 2015 Nov 26;16:1012. doi: 10.1186/s12864-015-2226-5 (PMC4661969; doi:10.1186/s12864-015-2226-5)
Supplement: Additional file 1: Table S1. — Treatments and developmental stages for transcriptome sampling and summary of the Py. haitanensis transcriptome. (DOCX 17 kb) [file 12864_2015_2226_MOESM1_ESM.docx]

Additional file 1: Table S1 Treatments and developmental stages for transcriptome sampling and summary of the *Py. haitanensis* transcriptome

| **Treatment/Development stages** | **Experimental conditions** |
| --- | --- |
| Conchocelis | 22°C, 30 μmol photons m^-2^ s^-1^, 12L:12D. Whole blades were harvested 6 hr after dawn |
| Conchosporangium | 28°C, 30μmol photons m^-2^ s^-1^, 10L:14D. Whole blades were harvested 6 hr after dawn |
| Blade, low light density | 20°C, 50 μmol photons m^-2^ s^-1^, 12L:12D. Whole blades were harvested 6 hr after dawn |
| Blade, high temperature | In sea water for 4 hr at 30°C, 1250 μmol photons m^-2^ s^-1^ |
| Blade, low temperature | In sea water for 4 hr at 10°C, 1250 μmol photons m^-2^ s^-1^ |
| Blade, hypo-osmotic treatment | In sea water diluted with equal amounts of distilled water for 4 hr at 20°C, 1250 μmol photons m^-2^ s^-1^ |
| Blade, hyperosmotic treatment | In sea water with the addition of 33 g/L NaCl for 4 hr at 20°C, 1250 μmol photons m^-2^ s^-1^ |
| Blade, dark treatment | In sea water for 4 hr at 20°C in darkness |
| Blade, nutrient limitation | Cultured for 6 d in seawater with no enrichment; 20°C, 1250 μmol photons m^-2^ s^-1^. The thalli began to turn green on the third day, indicating nitrogen limitation. Blades were harvested 3 hr after dawn |
| Blade, middle dehydrated | The thalli were air-dried for 1.5 hr at 20°C, 1250 μmol photons m^-2^ s^-1^. Whole blades harvested without any rehydration |
| Blade, severe dehydrated | The thalli were air-dried for 4 hr at 20°C, 1250 μmol photons m^-2^ s^-1^. Whole blades harvested without any rehydration |
| **Category** | **Number** |
| Total number of raw reads | 53,455,026 |
| Total number of clean reads | 47,764,168 |
| Clean bases | 4.78 G |
| Average read length | 100 |
| Q20 | 93.68% |
| Q30 | 83.78% |
| GC percentage | 64.93% |
| **Transcripts (unigenes)** | |
| **Transcripts Length** | **Number** |
| 200-500 bp | 20,427 (19,138) |
| 500-1k bp | 6,460 (5,544) |
| 1k-2k bp | 3,231 (2,551) |
| >2k bp | 1,720 (1,303) |
| Total number | 31,838 (28,536) |
| N50_Length | 956 (827) |
| Mean_Length | 657 (607) |
