# Supplementary figures and images for: Genome-wide expression profiles of Pyropia haitanensis in response to osmotic stress by using deep sequencing technology
Source: BMC Genomics. 2015 Nov 26;16:1012. doi: 10.1186/s12864-015-2226-5 (PMC4661969; doi:10.1186/s12864-015-2226-5)

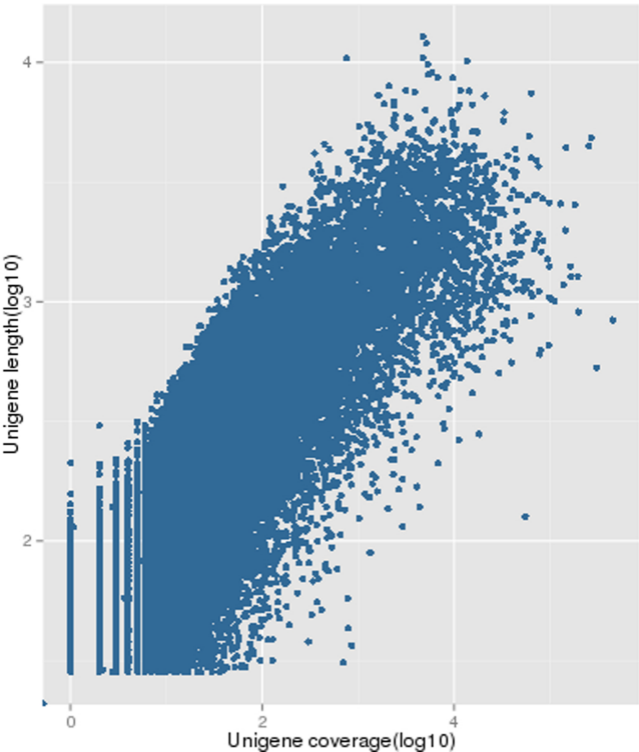

Supplement: Additional file 2: Figure S1. — Log-log plot showing the dependence of unigene lengths on the number of reads assembled into each unigene. (PDF 1390 kb) [file 12864_2015_2226_MOESM2_ESM.pdf]

CON\_1 vs CON\_2,  $R^2 = 0.936$

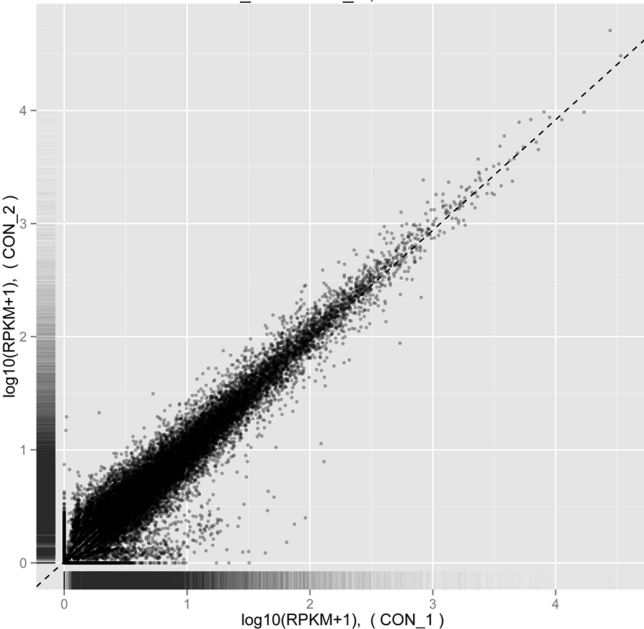

MWL\_1 vs MWL\_2,  $R^2 = 0.916$

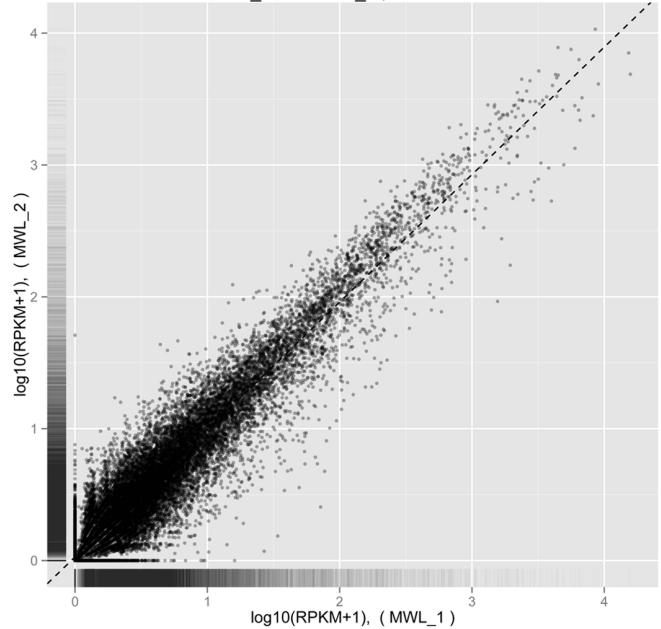

SWL\_1 vs SWL\_2,  $R^2 = 0.931$

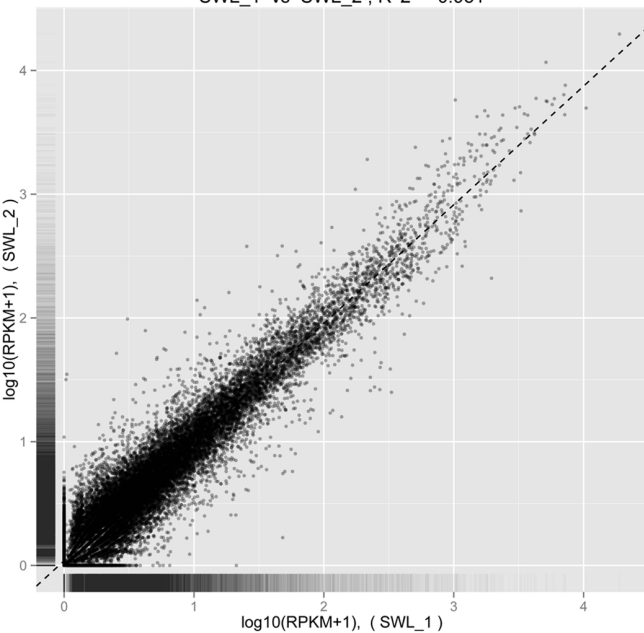

REH\_1 vs REH\_2,  $R^2 = 0.941$

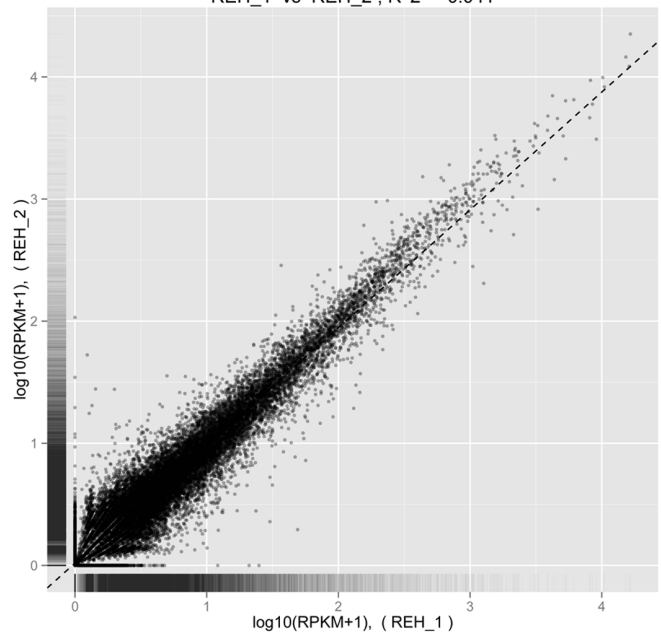

Supplement: Additional file 4: Figure S2. — Scatterplot showing the correlation of expression levels between two replicates. (PDF 2576 kb) [file 12864_2015_2226_MOESM4_ESM.pdf]
