# Supplementary material for: Genome-wide expression profiles of Pyropia haitanensis in response to osmotic stress by using deep sequencing technology
Source: BMC Genomics. 2015 Nov 26;16:1012. doi: 10.1186/s12864-015-2226-5 (PMC4661969; doi:10.1186/s12864-015-2226-5)
Supplement: Additional file 3: Table S2. — Summary of clean reads aligned uniquely to the reference transcriptome for each library. (DOCX 13 kb) [file 12864_2015_2226_MOESM3_ESM.docx]

Additional file 3: Table S2 Summary of clean reads aligned uniquely to the reference transcriptome for each library

| Sample name | Total reads | Total mapped | % mapped reads | No. unigenes hit |
| --- | --- | --- | --- | --- |
| MWL_1 | 13,050,154 | 12,670,756 | 97.09 | 19,843 |
| MWL_2 | 14,240,476 | 13,919,320 | 97.74 | 20,030 |
| SWL_1 | 13,279,452 | 12,851,226 | 96.78 | 20,464 |
| SWL_2 | 14,781,653 | 14,329,268 | 96.94 | 20,459 |
| REH_1 | 14,168,609 | 13,766,327 | 97.16 | 20,495 |
| REH_2 | 11,830,575 | 11,496,294 | 97.17 | 20,534 |
| CON_1 | 15,041,472 | 14,564,468 | 96.83 | 21,954 |
| CON_2 | 14,827,844 | 14,398,493 | 97.10 | 20,995 |
