# Supplementary material for: Genome-wide expression profiles of Pyropia haitanensis in response to osmotic stress by using deep sequencing technology
Source: BMC Genomics. 2015 Nov 26;16:1012. doi: 10.1186/s12864-015-2226-5 (PMC4661969; doi:10.1186/s12864-015-2226-5)
Supplement: Additional file 13: Table S11. — Differentially expressed genes encoding ABC transporters between REH and CON based on KO database annotation. (DOCX 15 kb) [file 12864_2015_2226_MOESM13_ESM.docx]

Additional file 13: Table S11 Differentially expressed genes encoding ABC transporters between REH and CON based on KO database annotation

| **Unigene ID** | **Unigene**  **length (bp)** | **REH^a^** | **CON^b^** | **Log_2_ fold change^c^** | **Adjusted P-value^d^** | **KO description** |
| --- | --- | --- | --- | --- | --- | --- |
| comp16736_c0 | 1165 | 212.3201650325 | 844.616059332666 | -1.9921 | 0.020439 | ATP-binding cassette, subfamily B (MDR/TAP), member 1 |
| comp16736_c2 | 574 | 57.2488399526303 | 183.081459358829 | -1.6772 | 0.004913 | ATP-binding cassette, subfamily B (MDR/TAP), member 1 |
| comp8118_c0 | 2599 | 599.203487951028 | 1611.68806221905 | -1.4275 | 0.002689 | ATP-binding cassette, subfamily G (WHITE), member 2 |
| comp18304_c0 | 1946 | 718.902979707798 | 1743.23280932196 | -1.2779 | 0.0099477 | ATP-binding cassette, subfamily B (MDR/TAP), member 4 |
| comp21051_c0 | 2872 | 672.323231363948 | 1516.52563715845 | -1.1735 | 0.022202 | mitochondrial ABC transporter ATM |

^a^ Read counts of differentially expressed genes encoding ABC transporters under rehydration (REH).

^b^ Read counts of differentially expressed genes encoding ABC transporters under normal conditions (CON).

^c^ Log_2_ fold change (REH/CON).

^d^ Differentially expressed genes with a adjusted P-value < 0.05 are shown.
