# Supplementary material for: Genome-wide expression profiles of Pyropia haitanensis in response to osmotic stress by using deep sequencing technology
Source: BMC Genomics. 2015 Nov 26;16:1012. doi: 10.1186/s12864-015-2226-5 (PMC4661969; doi:10.1186/s12864-015-2226-5)
Supplement: Additional file 14: Figure S3. — (A) Nucleotide and deduced amino acid sequence of cDNA encoding 1,4-alpha-glucan branching enzyme (PhSBE). Nucleotide residues are numbered in the 5’ to 3’ direction. Amino acid residues are numbered in the N- to C-terminal direction. The start codon is marked in red color font. An asterick denotes the stop codon. (B) Genomic sequence structure of the PhSBE ORF (5’ to 3’ direction). 1 (arrow) represents start site of translation, 2767 represents end site of translation. Exon 1 (1-28), exon 2 (320-401) and exon 3 (610-2767) are depicted as red boxes. Intron 1 (29-319) and intron 2 (402-609) are depicted as narrow blue boxes. (PDF 170 kb) [file 12864_2015_2226_MOESM14_ESM.pdf]

1 ATGGCGGAGAAGGATGGCCACTTTGATGCTTGGAAGGATAAGAAGGATGGCACCCAGGTG
1 M A E K D G H F D A W K D K K D G T Q V

61 ATTGCCGCCGACCGCTACCTGGAGCCTTATGCGGATTCGCTGCGGTATCGCTTCACCAAG
21 I A A D R Y L E P Y A D S L R Y R F T K

121 TACAACGAGATCAAAAACGCCATCGAGGAGAGCGAAGGCGGCCTGGGAAAGTTTGCGCAG
41 Y N E I K N A I E E S E G G L G K F A Q

181 TCCTACAAGTCGTTTGGCCTGCACGCTGTAGAGGGGGGCGTTGAGTACCGTGAGTGGGCT
61 S Y K S F G L H A V E G G V E Y R E W A

241 CCTGGCGCTCAGTCCGTGTCTGTCTTTGGCGACTTCAACGGCTGGAACCGGAACTCGCAT
81 P G A Q S V S V F G D F N G W N R N S H

301 CAGCTGACGCGTGGGGAGTTTGGGATTTGGACGACCACCATTCCGGACAATGAGGACGGT
101 Q L T R G E F G I W T T T I P D N E D G

361 TCCCCGGCGGTGCCGCACGGCTCCAAGGTGAAGGTGTGCATTGTGACTCCCGGCGGGATG
121 S P A V P H G S K V K V C I V T P G G M

421 CACCTGGATCGGAACCCGGCGTGGGCCACGTACCTCATCCAGAACCCGTCGACGCTCCTG
141 H L D R N P A W A T Y L I Q N P S T L L

481 TTTGATACGGTGTTTTGGAACCCGCCGGAGGAGCACAAGTACCAGTGGAAGCACCAGAAG
161 F D T V F W N P P E E H K Y Q W K H Q K

541 CACCCCCCGGCCCCAGAGTGCATGCGCATCTACGAATGCCACGTCGGGATGGGCTCGGCC
181 H P P A P E C M R I Y E C H V G M G S A

601 GACCCCAAGGTGGGCACCTACGACGAATTCACCGACAACATCCTGCCGCGGATTAAGGAC
201 D P K V G T Y D E F T D N I L P R I K D

661 CTGGGCTTCACAGCCATCCAGATTATGGCCATCATGGAGCATGCGTACTACGGTTCTTTT
221 L G F T A I Q I M A I M E H A Y Y G S F

721 GGTTACCACGTCACCAACTTTTTTGCCATTTCTTCTCGGAGCGGCGATCCAGAAGGGCTC
241 G Y H V T N F F A I S S R S G D P E G L

781 AAGCGCTTGATTGACACGGCGCATGGCATGGGGCTTGTTGTCCTGATGGATGTCGTTCAC
261 K R L I D T A H G M G L V V L M D V V H

841 AGCCACGCCTCCTCCAACTCGATGGATGGGATCAACCAGTTTGATGGCACCGACCACCAG
281 S H A S S N S M D G I N Q F D G T D H Q

901 TACTTCCATGAGGGCGAGCGCGGTCGGCACTCGCTGTGGGACTCGCGGCTGTTCAACTAT
301 Y F H E G E R G R H S L W D S R L F N Y

961 GGGCAGTGGGAGGTTATTCGCTTCCTGCTTTCCAACCTGCGCTGGTACATGGAGGAGTAC
321 G Q W E V I R F L L S N L R W Y M E E Y

1021 CACTTTGACGGGTTCCGCTTTGACGGGGTTACGTCCATGCTGTACAAGCACCACGGCATC
341 H F D G F R F D G V T S M L Y K H H G I

1081 CAGGTGCAGTTCTCGGGCGACTACCGCGAGTACTTTGGCATGCACGTCGATGTTGACGCG
361 Q V Q F S G D Y R E Y F G M H V D V D A

1141 TGCGTCTACCTCATGTTGGCGAACGACCTTGTGCGGCAGGTGAACCCGGATTCGGGAATC
381 C V Y L M L A N D L V R Q V N P D S G I

1201 ACCATTGCTGAGGACGTGTCGGGCATGCCTACCGTGTGCCGCCCGGTCGTGGAGGGCGGG
401 T I A E D V S G M P T V C R P V V E G G

1261 TTGGGCTTTGACTACCGGTTGGGCATGTCTGTGCCTGACAAGTGGATTGAGCTGCTGTCC
421 L G F D Y R L G M S V P D K W I E L L S

1321 AAGGAGAAGGACGAGGCGTGGAACATGGGCAATATTGCGTTCACCCTGACCAACCGGCGT
441 K E K D E A W N M G N I A F T L T N R R

1381 TGGAACGAGGCCACCATTGGCTACGCTGAGTCCCACGACCAGGCGCTGGTGGGGGATAAG
461 W N E A T I G Y A E S H D Q A L V G D K

1441 ACGCTGGCTTTTTGGCTAATGGACGCGGCTATGTACACTAGCATGGGGATGGATCAACAA
481 T L A F W L M D A A M Y T S M G M D Q Q

1501 TCCCCGGTGGTGGAGCGCGGTATCGCGCTGCACAAAATGATCCGGCTGATCTCTTATGGC
501 S P V V E R G I A L H K M I R L I S Y G

1561 TTGGCTGGTGAAGGCTACCTGACCTTCATGGGCAACGAGTTTGGTCACCCCGAGTGGGTG
521 L A G E G Y L T F M G N E F G H P E W V

1621 GACTTCCCTCGGGCCGGCAACGGTTTTTCGTACGAGAAGGCGCGGCGGCGGTGGGACCTG
541 D F P R A G N G F S Y E K A R R R W D L

1681 GCGGACAACAAGGGCCTCCGCTACTCCCATATGCAGCTGTGGGAGAAGCTGATGCACGAG
561 A D N K G L R Y S H M Q L W E K L M H E

1741 CTTGAGACCAGCCACTTCTTTTGCCGCAAGGCGGTGCACCAGTACGTTGTGCTTGCGCAT

581 L E T S H F F C R K A V H Q Y V V L A H

1801 GACCAGGACAAGGTGGTGGCGTTTGAGAAGGGAGACCGGCTGCTGTTTGTCTTCAACTTC
601 D Q D K V V A F E K G D R L L F V F N F

1861 CATCCTACCAAGTCGTACACCGATTACCGCATCGGCACTCACTGGGGCGGAAAGTACCGC
621 H P T K S Y T D Y R I G T H W G G K Y R

1921 CTGGTGCTAGATTCGGACGGGACCAACGTGGGTGGCCAAGGCCGGGTACACTGGCACGTG
641 L V L D S D G T N V G G Q G R V H W H V

1981 GTGCACCGGACCTCCAGCTCGCCATGGCAGTCGCGGTCTCACTCGCTCCAGTTGTACCTG
661 V H R T S S S P W Q S R S H S L Q L Y L

2041 CCGTCGCGCACCTGCCAAGTGTATCATTGCTTTGAGCTGGACTCGAAGGCGGAGGAGGCA
681 P S R T C Q V Y H C F E L D S K A E E A

2101 GCGCACAAGGCGGCACCTGCGGCTGCGGCGGCGACTACAGCAGAGACTGTCGCTGAGGTT
701 A H K A A P A A A A A T T A E T V A E V

2161 GACGCGGCTAAGGCGGCGCCCGCAGCCAAGACTGCTGCTTCCCCAACAGACGCGGCTGCC
721 D A A K A A P A A K T A A S P T D A A A

2221 CCGGAAGAGGCTGTGAAGGCAGATGGAGCCAAGAAGGCCTCTGCCTAA
741 P E E A V K A D G A K K A S A *

Additional file 14: Figure S3 A Nucleotide and deduced amino acid sequence of cDNA encoding 1,4-alpha-glucan branching enzyme (*Ph*SBE). Nucleotide residues are numbered in the 5’ to 3’ direction. Amino acid residues are numbered in the N- to C-terminal direction. The start codon is marked in red color font. An asterick denotes the stop codon.

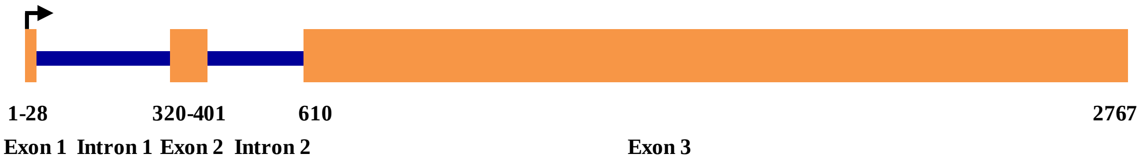


Additional file 14: Figure S3 B Genomic sequence structure of the *Ph*SBE ORF (5’ to 3’direction). 1 (arrow) represents start site of translation, 2767 represents end site of translation. Exon 1 (1-28), exon 2 (320-401) and exon 3 (610-2767) are depicted as red boxes. Intron 1 (29-319) and intron 2 (402-609) are depicted as narrow blue boxes.
