# Supplementary material for: Genome-wide expression profiles of Pyropia haitanensis in response to osmotic stress by using deep sequencing technology
Source: BMC Genomics. 2015 Nov 26;16:1012. doi: 10.1186/s12864-015-2226-5 (PMC4661969; doi:10.1186/s12864-015-2226-5)
Supplement: Additional file 15: Figure S4. — Multiple sequence alignment of 1,4-alpha-glucan branching enzymes from different species. 1,4-alpha-glucan branching enzymes were chosen from different species and aligned by DNAMAN. Sequence in frame means Alpha-amylase_C domain. The accession numbers of these sequences were showed as follows: Chondrus crispus (XP_005716136.1), Gracilaria gracilis (AAB97471.1), Cyanidioschyzon merolae strain 10D (XP_005536101.1), Galdieria sulphuraria (XP_005703889.1), Arabidopsis thaliana (NP_195985.3), Zea mays (NP_001105316.1) and Oryza sativa (BAA82828.1). (PDF 12907 kb) [file 12864_2015_2226_MOESM15_ESM.pdf]

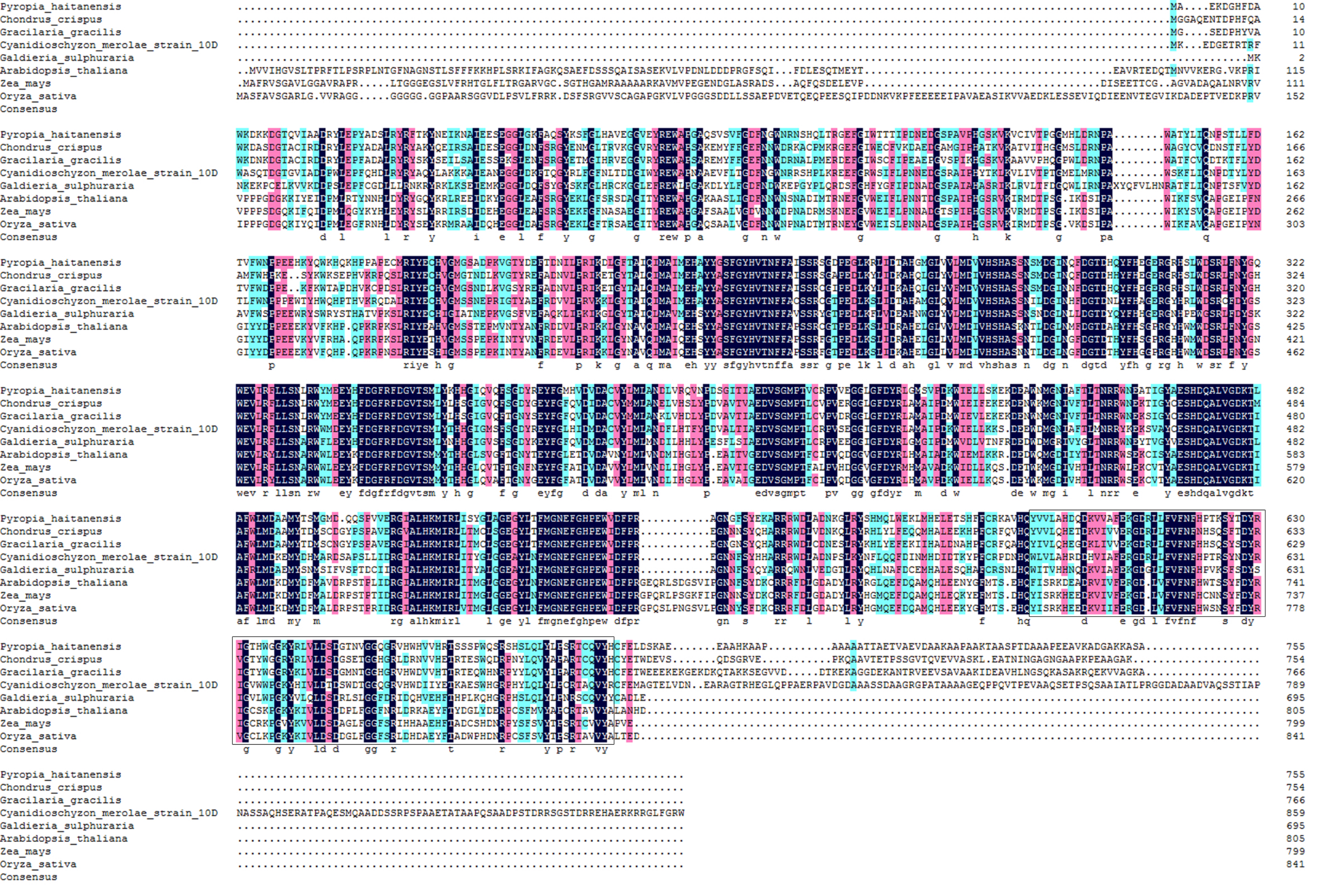
Pyropia_haitanensis
Chondrus_crispus
Gracilaria_gracilis
Cyanidioschyzon_merolae_strain_10D
Galdieria_sulphuraria
Arabidopsis_thaliana
Zea_mays
Oryza_sativa
Consensus
