# Supplementary material for: Genome-wide expression profiles of Pyropia haitanensis in response to osmotic stress by using deep sequencing technology
Source: BMC Genomics. 2015 Nov 26;16:1012. doi: 10.1186/s12864-015-2226-5 (PMC4661969; doi:10.1186/s12864-015-2226-5)
Supplement: Additional file 16: Table S12. — Primers used in qRT-PCR for validating differentially expressed genes. (DOCX 14 kb) [file 12864_2015_2226_MOESM16_ESM.docx]

Additional file 16: Table S12 Primers used in qRT-PCR for validating differentially expressed genes

| Gene | Gene ID | Primer sequence 5’→3’ forward/reverse | Amplicon size (bp) |
| --- | --- | --- | --- |
| 18S ribosomal RNA |  | AGTTAGGGGATCGAAGACGA/ CAGCCTTGCGACCATACTC | 153 |
| Ubiquitin-conjugating enzyme |  | TCACAACGAGGATTTACCACC/ GAGGAGCACCTTGGAAACG | 107 |
| 1,4-alpha-glucan branching enzyme | comp17143_c0 | GGGACTCAGCGTAGCCAAT/ CTGTGCCTGACAAGTGGAT | 126 |
| Pyruvate kinase | comp7242_c0 | GCACTGACCGAAAAGGAT/ CCGAGACTGCCAGGATAT | 208 |
| Malate dehydrogenase | comp15628_c0 | TGGTGTGGAATTGGAATCGG/ AAAGGCGAAGCGGGTCAA | 156 |
| Omega-6 fatty acid desaturase | comp19838_c0 | TTCTGGGTGAGGAGTTTCC/ CGAGCACAATGGCATTCTC | 174 |
| Delta6-fatty acid desaturase | comp15419_c0 | CGGTGACATTGTGCTAACG/ CGCTGGTAGTGCCATCAATA | 161 |
| Delta-6 fatty acid elongase | comp21618_c0 | TGGCAAACCAGTAGGACAG/ GCTCACCTGGATCTTTACCT | 137 |
